# Genomic analysis of a new heterotic maize group reveals key loci for pedigree breeding

**DOI:** 10.3389/fpls.2023.1213675

**Published:** 2023-08-11

**Authors:** Zhiyong Li, Chunhui Li, Ruyang Zhang, Minxiao Duan, Hongli Tian, Hongmei Yi, Liwen Xu, Fengge Wang, Zi Shi, Xiaqing Wang, Jidong Wang, Aiguo Su, Shuai Wang, Xuan Sun, Yanxin Zhao, Shuaishuai Wang, Yunxia Zhang, Yuandong Wang, Wei Song, Jiuran Zhao

**Affiliations:** Beijing Key Laboratory of Maize DNA Fingerprinting and Molecular Breeding, Maize Research Institute, Beijing Academy of Agriculture and Forestry Sciences, Beijing, China

**Keywords:** X lines, heterotic group, ICRs, SSWs, yield-related genes

## Abstract

Genome-wide analyses of maize populations have clarified the genetic basis of crop domestication and improvement. However, limited information is available on how breeding improvement reshaped the genome in the process of the formation of heterotic groups. In this study, we identified a new heterotic group (X group) based on an examination of 512 Chinese maize inbred lines. The X group was clearly distinct from the other non-H&L groups, implying that X × HIL is a new heterotic pattern. We selected the core inbred lines for an analysis of yield-related traits. Almost all yield-related traits were better in the X lines than those in the parental lines, indicating that the primary genetic improvement in the X group during breeding was yield-related traits. We generated whole-genome sequences of these lines with an average coverage of 17.35× to explore genome changes further. We analyzed the identity-by-descent (IBD) segments transferred from the two parents to the X lines and identified 29 and 28 IBD conserved regions (ICRs) from the parents PH4CV and PH6WC, respectively, accounting for 28.8% and 12.8% of the genome. We also identified 103, 89, and 131 selective sweeps (SSWs) using methods that involved the π, Tajima’s D, and CLR values, respectively. Notably, 96.13% of the ICRs co-localized with SSWs, indicating that SSW signals concentrated in ICRs. We identified 171 annotated genes associated with yield-related traits in maize both in ICRs and SSWs. To identify the genetic factors associated with yield improvement, we conducted QTL mapping for 240 lines from a DH population (PH4CV × PH6WC, which are the parents of X1132X) for ten key yield-related traits and identified a total of 55 QTLs. Furthermore, we detected three QTL clusters both in ICRs and SSWs. Based on the genetic evidence, we finally identified three key genes contributing to yield improvement in breeding the X group. These findings reveal key loci and genes targeted during pedigree breeding and provide new insights for future genomic breeding.

## 1 Introduction

The total maize grain yield increased 12-fold during the past six decades (Li, 2009), but by 2050, the total demand for cereal grains is projected to increase by 56%, with the demand for maize expected to increase by 45% (Hubert et al., 2010). Therefore, improving yield is a major objective for maize producers. Genome-wide analyses of diverse inbred lines have helped characterize the genetic basis of crop improvement and domestication (Lai et al., 2010; Hufford et al., 2012; Jiao et al., 2012; Unterseer et al., 2016); however, we know little about the formation of heterotic groups during breeding. Research regarding the process underlying genetic enhancements may further characterize the key genomic regions for breeding, thereby providing the basis for improved maize breeding, enhanced gene cloning, and molecular breeding by design.

Previous studies investigated the genome-wide changes that occurred during maize breeding. For example, Wu et al. (2014) used a high-throughput microarray to study 367 inbred lines, which detected conserved genetic regions in specific subgroups. Additionally, on the basis of an identity-by-descent (IBD) analysis, they identified 15 conserved regions transmitted from Huangzaosi to its descendants. In another investigation, a comparison between 41 unselected lines from an Iowa Stiff Stalk Synthetic (BSSS) population and 21 highly selected lines developed by modern commercial breeding programs revealed significant decreases in tassel size and weight (Gage et al., 2018). Using 14 lines derived from Huangzaosi, Zhang et al. (2018) identified 52 common regions and 38 regions co-localized with quantitative trait loci (QTLs) for important agronomic traits. Li et al. (2019) analyzed 40 Huangzaosi-related lines by resequencing and detected 862 IBD conserved regions (ICRs) that were highly consistent with selective sweep regions. They also determined that yield-related genes/QTLs are enriched in these regions. Wang et al. (2020) identified more than 1,800 genomic regions carrying genes related to traits targeted for selection in modern breeding programs as well as 160 loci underlying adaptive agronomic phenotypes. They also validated high-confidence candidate genes using selection scan methods and genome-wide association mapping. Other crops have also been thoroughly investigated, including rice (Zhou et al., 2016). However, the maize genetic improvement process associated with pedigree breeding involving generations derived from F_1_ lines remains relatively uncharacterized, with only limited research on genome-wide genetic improvements during breeding (Wang et al., 2020).

In recent years, breeders have produced new maize lines using the new germplasm X1132X, which was introduced from the USA, under strict selection conditions, including high planting densities, large populations, location shifts, and exposures to high stress levels. Many elites inbred lines have been generated, including Jing724, Jing725, Jing464, and JingMC01. A new pattern of heterosis was investigated because X lines exhibit strong heterosis with Huangzaosi improved lines (HILs). On the basis of this pattern, a series of new hybrids has been bred, including Jingke968 (Jing724 × Jing92), Jingnongke728 (JingMC01 × Jing2416), Jingke665 (Jing725 × Jing92), and NK718 (Jing464 × Jing2416). So far, dozens of approved hybrids that are widely cultivated have been bred using X lines (**Supplementary Table 1**). However, we know little about the genome-wide changes during the breeding of improved X lines.

This study reveals the genome-wide genetic improvements that occurred during the breeding of X lines. Based on an analysis of 512 Chinese maize inbred lines, a new heterotic group (X group) was identified. Seventeen core inbred lines, including 15 elite X lines and two X line parents (**Supplementary Table 2**), were selected for whole-genome resequencing. This genome sequencing data enabled us to clarify genomic recombinations and identify key genome regions that occurred during pedigree breeding. Additionally, multiple annotation methods were employed to identify yield-related loci and genes in the target regions that are relevant for modern maize breeding. In addition, a QTL mapping experiment was conducted for a doubled haploid (DH) population (PH4CV × PH6WC, which are the parents of X1132X), comprising 240 lines in multiple environments. Lastly, three key genes contributing to yield improvement in the breeding of X lines were identified. These findings not only provide important insights into how genome-wide changes occurred during the breeding improvement of a new heterotic group, but also identify the key genes using multiple genetic methods.

## 2 Materials and methods

### 2.1 Plant materials and maizeSNP3072 SNP genotyping

In this study, 512 maize inbred lines and a DH population consisting of 240 lines were used for genetic analyses. The maize panel contained important heterotic groups commonly used for maize breeding in China, including HIL, Lucia Red Cob (LRC), Domestic Reid (DR), P group, and X group. The inbred lines in these groups reflect the genetic diversity of the germplasm resources used for maize breeding in China. The DH population was derived from a cross between PH4CV and PH6WC.

For all lines, leaf tissue samples were obtained from a pool of at least 30–50 individuals for a DNA extraction according to a CTAB procedure (Murray and Thompson, 1980). All lines were genotyped by the Illumina MaizeSNP3072 assay (Tian et al., 2015). Raw data were obtained by scanning the chip for hybridization signals using the Illumina iScan instrument. The genotyping data for each sample were analyzed using the GenomeStudio software (version 2011.1) (Illumina and GoldenGate data). The single nucleotide polymorphisms (SNPs) with low scores, ambiguous clusters, and a missing data rate greater than 50% were eliminated. The remaining 2,869 high-quality SNPs were used for subsequent genetic analyses.

### 2.2 Genome library construction and resequencing

Resequencing of the genome was conducted on 15 representative X lines and two parental lines. All of the 15 selected X lines have bred many approved varieties (**Supplementary Table 1**). We constructed paired-end sequencing libraries from at least 5 µg extracted genomic DNA according to the manufacturer’s instructions (Illumina). The libraries were sequenced using the Illumina NovaSeq 6000 or HiSeq 2500 platforms at Berry Genomics (Beijing, China) to generate 150-bp or 125-bp short reads at each end.

### 2.3 Genome read mapping and variant calling

Low-quality reads and adapter sequences were removed using fastp (Chen et al., 2018). The clean reads were mapped to the B73 reference sequence (version AGPv3) with the default parameters of the BWA software, after which the alignment results were merged and indexed as BAM files (Li and Durbin, 2009; Li et al., 2009). To minimize the number of mismatched bases for SNP and insertion/deletion (InDel) calling, all reads were further filtered to eliminate unmapped and non-unique reads for a sequence alignment using the IndelRealigner package. The SNP and InDel calling was based on an alignment using the Genome Analysis Toolkit (GATK; version 3.6-0-g89b7209) (McKenna et al., 2010) and the Picard package (version 1.119). Specifically, SNP and InDel calling for each sample was performed independently using the HaplotypeCaller package in GATK. The SNP and InDel calling at the population level (i.e., concurrently for all sequenced genomes) was performed using the GenotypeGVCFs package in the GATK pipeline, with a minimum phred-scaled confidence threshold for variant calling of 60, a mapping quality score > 40, and a sequence depth for genotypes > 2 in every sample.

### 2.4 Population and phylogenetic analyses

The ADMIXTURE program (Alexander et al., 2009) was used to assess the genetic relationships among the X lines. The ancestor populations were inferred by K values from 2 to 8. A neighbor-joining phylogenetic tree was constructed using the Treebest software (version 1.9.2) (Vilella et al., 2009). Additionally, a principal component analysis (PCA) was completed using the GCTA software (version 1.26.0) (Yang et al., 2011).

### 2.5 Identity-by-descent analyses

The IBD segments in the X lines derived from the parents were identified with IBDseq (Browning and Browning, 2013), with the LOD score for detecting IBD segments set at 3 and the end rimming of IBD segments set at 2.5. The number of markers in the sliding window used for detecting correlated markers was set at 4,000 and the maximum allele error rate was set at 0.0001. Finally, we used the principle of minimum recombination times to determine the attribution gap.

### 2.6 Genome scanning for selection-related signals

Evidence of selection across the genome during the genetic improvement of X lines was revealed using π, Tajima’s D (Tajima, 1989), and CLR (Nielsen et al., 2009) methods. We used the CLR value to detect selective sweeps in X line genomes with SweeD (Pavlidis et al., 2013), which identifies signals of selective sweeps based on significant deviations from the neutral site frequency spectrum (Li et al., 2017). The π and Tajima’s D values were calculated using Variscan (Hutter et al., 2006). All three values were calculated with a 10-kb sliding window across 10 chromosomes using high-quality SNPs. Finally, we calculated the mean likelihood score in 100-kb sliding windows across the genome. The highest π, Tajima’s D, and CLR values, accounting for 10% of the genome, were used to select regions (Xie et al., 2015). Additionally, adjacent selected windows were grouped into a single region to represent a selective sweep region.

### 2.7 Phenotyping

We assessed the agronomic and yield-related characteristics of 240 DH lines and elite X lines. To conduct phenotyping, 240 DH lines were planted in three environmental conditions in 2015, each with two replicates. The lines were planted in Yujiawu and Xiaotangshan in the spring of 2015. Specifically, the population was planted in Yujiawu at high temperature environmental in the summer of 2015. The experimental design involved two replicates for each DH line in each environment, utilizing a randomized complete block design. Each DH line was allocated an entire row, with a row length of 5 m, row spacing of 60 cm, and a plant spacing of 27.5 cm.

The lines were analyzed regarding the following 10 traits: hundred grain weight (HGW), kernel number per ear (KNPE), ear weight (EW), bare top length (BTL), ear length (EL), water content (WC), volume weight (VW), kernel number per row (KNPR), kernel weight per ear (KWPE), and rows per ear (RPE). The quantitative traits were evaluated based on the mean of five independent measurements. Regarding the weight-related traits (i.e., HGW, VW, and KWPE), we used the WC to correct the data. Specifically, values for all traits were adjusted based on a WC of 13% for the subsequent QTL analyses.

### 2.8 QTL mapping

Genetic maps for the DH population were constructed using the nnTwoOpt algorithm for ordering and the ripple-SARF algorithm in IciMapping (version 4.2) (Meng et al., 2015). The genetic distance between markers was determined in centimorgans (cM) using the Kosambi function. The QTL mapping was performed using the default setting of BIP (QTL mapping in bi-parental populations) in IciMapping (version 4.2) (Meng et al., 2015), with a 0.5 cM scanning step. Regarding the marker stepwise regression, the probability level of variables in and out of the model was 0.001. An LOD score of 2.5 was set as the threshold for QTL mapping and for estimating QTL effects.

## 3 Results

### 3.1 Population structure of the Chinese maize panel

**Figure 1:**
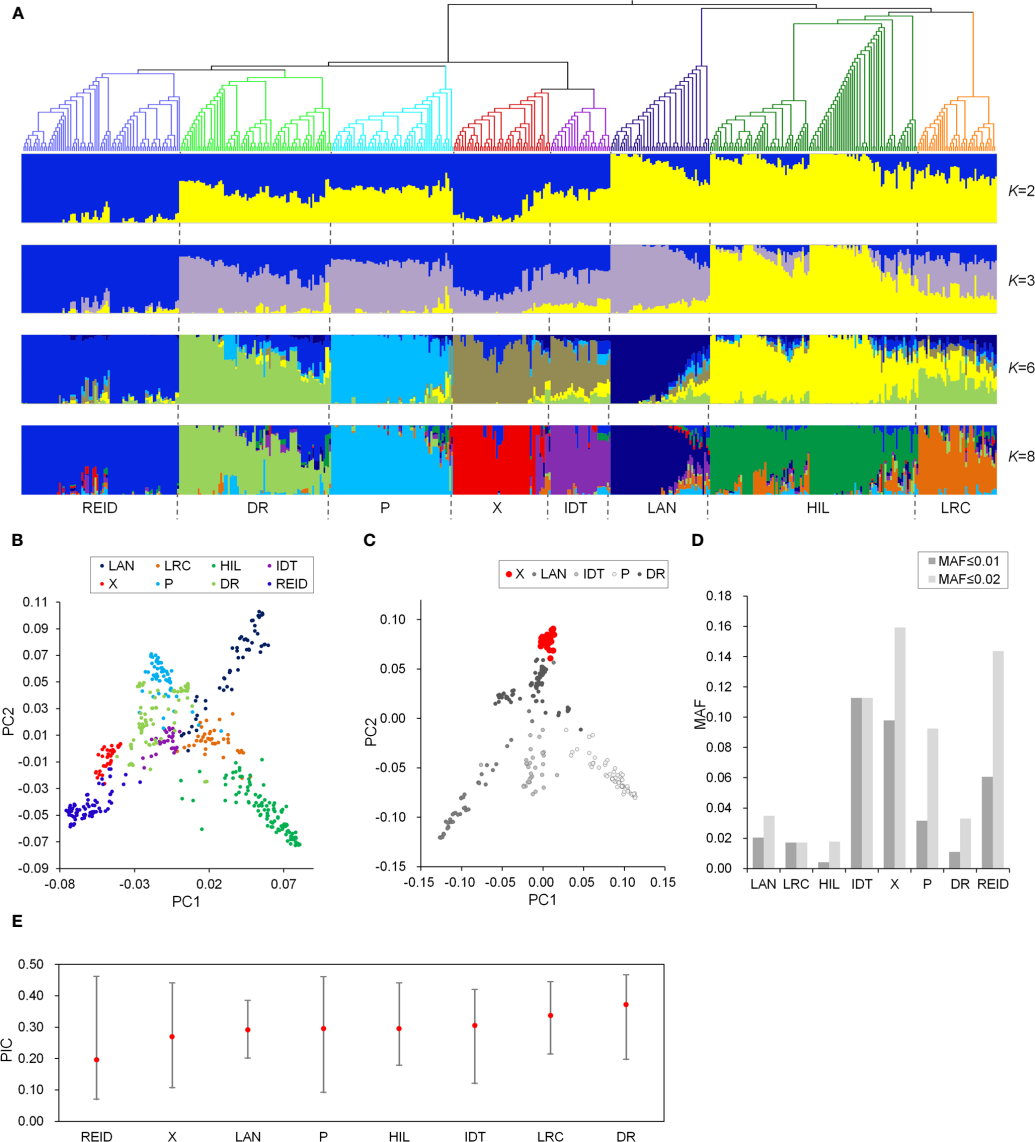
Phylogenetic and population structure analyses of a Chinese maize panel. **(A)**, Neighbor-joining phylogenetic tree of the maize panel and model-based clustering (K = 2, 3, 6, and 8). Branch colors indicate different subgroups (matching the colors in a). **(B)**, Principal component analysis of the maize panel. PC1, first principal component; PC2, second principal component. **(C)**, Principal component analysis of five genetically related subgroups. (**D)**, Proportion of MAF less than 0.01 and 0.02 among different subgroups. **(E)**, PIC for different subgroups.

A maize panel comprising 512 diverse inbred lines was used in this study. The maize panel included lines that have been used or were recently improved in Chinese maize breeding programs. An examination of the population structure using a fixed group number K of 1 to 8 revealed distinct relationships between subgroups. When K = 2 or 3, the inbred lines derived from Chinese landraces, such as Huangzaosi and Dan340, were grouped together (**Figure 1A**). The other inbred lines, which formed the introduced germplasm group, were mainly the introduced inbred lines and lines improved from the introduced hybrids. When K = 8, the maize panel was further divided into the following eight subgroups: LRC, HIL, DR, P, X, Iodent (IDT), Lancaster (LAN), and Reid. Phylogenetic analyses and PCA results revealed a consistent relationship among population structures, and the eight subgroups were genetically related (**Figure 1A, B** **)**. Moreover, the X group was identified as a new and elite subgroup, which included lines that were selected by pedigree breeding from X1132X hybrids (Zhao et al, 2017; Zhao et al, 2018). The PCA of five genetically related subgroups (**Figure 1C**) indicated the X group clearly differs from the other subgroups. The X group population had relatively low levels of genetic diversity, but a relatively high proportion of rare alleles (**Figures 1D, E**).

### 3.2 Genome structure of X group inbred lines

**Figure 2:**
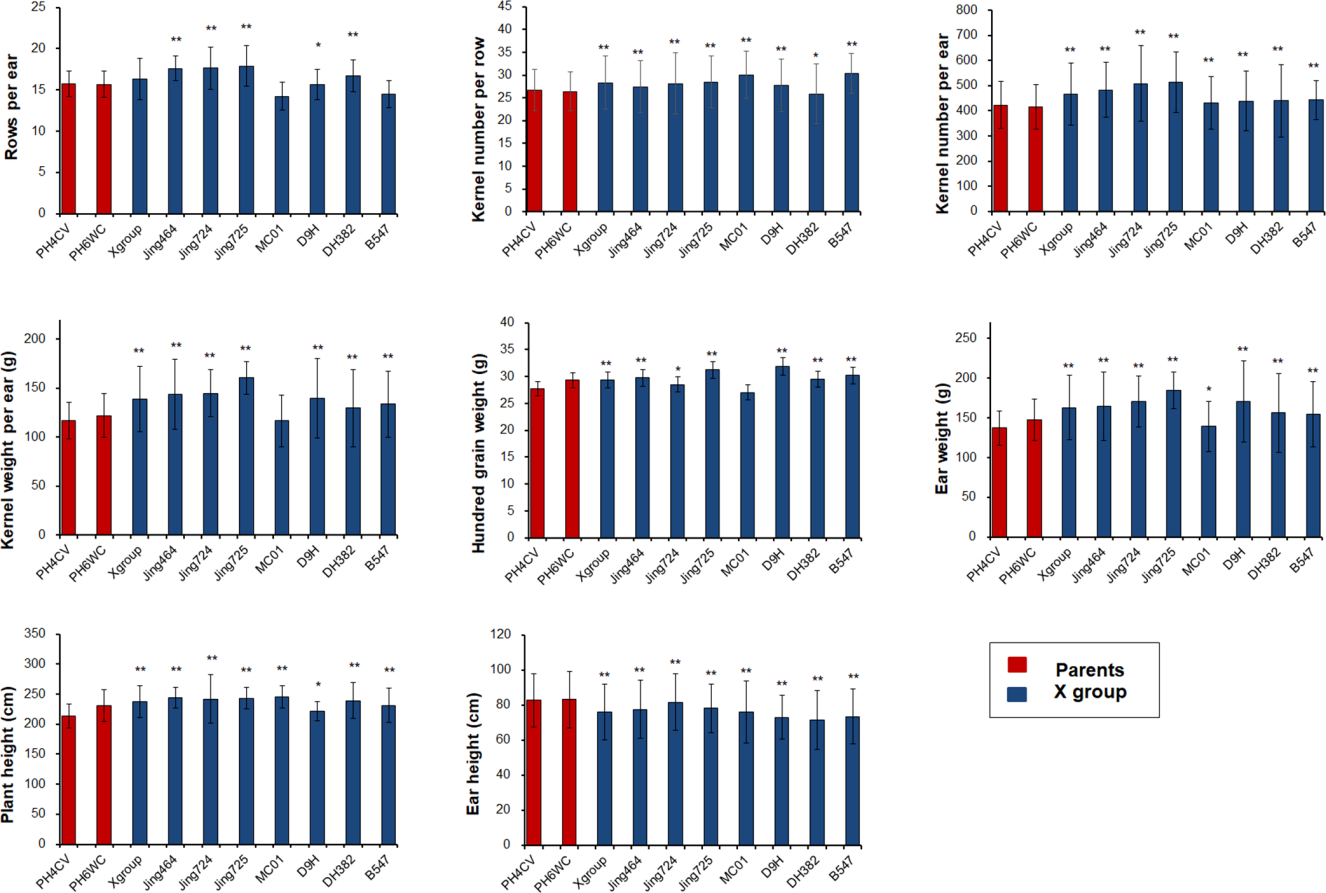
Improvement of important agronomic traits in the X lines and their parents. Significance levels: *P<0.05; **P < 0.01.

We selected 17 inbred lines (15 elite X lines and two parents of X lines) for an analysis of agronomic traits. Substantial improvements were detected for many agronomic traits among the X lines (e.g., increased plant height and decreased ear height). Most of the yield-related traits were considerably better in the X lines than in the parental lines, including RPE, KNPR, KNPE, KWPE, HGW, and EW. Thus, the primary improvement of the X group during breeding was enhanced yield-related traits (**Figure 2**).

To clarify the genome structure and identify the artificially selected genome regions in the X lines, we generated whole-genome sequences of 17 representative inbred lines (15 elite X lines and the two parents), with an average sequence coverage of 17.35× (**Supplementary Table 2**). On average, 90.1% of the reads were mapped to the genome. The sequences of all inbred lines covered 81.5%–91.3% of the whole genome, with an average of 88.0%. A total of 36.5 M raw SNPs were identified. Subsets of these data were filtered for the following analysis.

**Figure 3:**
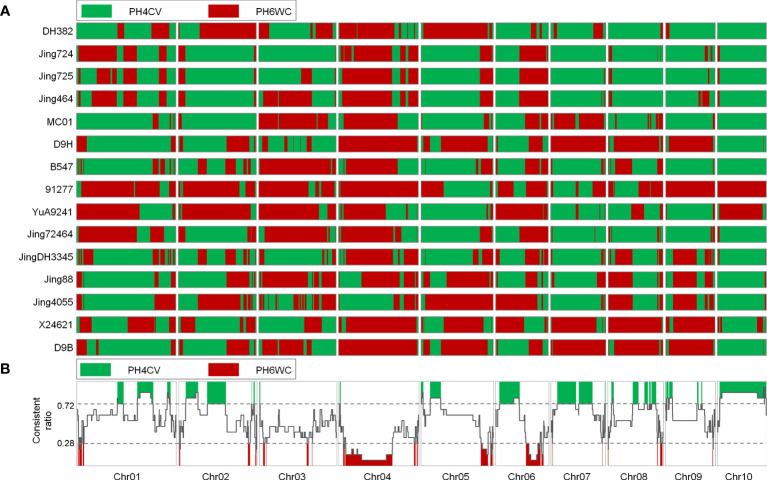
**(A)**, Genomic IBD regions in 15 elite X lines from two parents and the conserved genomic regions. Green and red genomic regions are derived from PH4CV and PH6WC, respectively. **(B)**, the line chart indicates the consistently retained PH4CV IBD regions in 15 elite X lines.

We analyzed the IBD segments transferred from the two parents to the X lines. Many complementary, long, and continuous IBD segments were identified in the two parents, suggesting that all X lines were derived from these two parents (**Figure 3A**). Overall, the ratio of the consistently retained PH4CV IBD segments to the consistently retained PH6WC IBD segments ranged from 0.234 to 0.727 (**Supplementary Figure 1**). More X lines have a high ratio indicated these inbred lines were more genetically related to PH4CV than to PH6WC (**Supplementary Figure 2**). From the whole genome distribution of IBD, we detected some genomic regions in which almost all of the IBD segments from the two parents were retained in the X lines (**Figure 3B**). 29 and 28 ICRs were detected from the parents PH4CV and PH6WC, accounting for 28.8% and 12.8% of the total genome, respectively. We speculated that these ICRs contained favorable alleles inherited from the two parents.

### 3.3 Genomic imprints of selection during X group breeding

**Figure 4:**
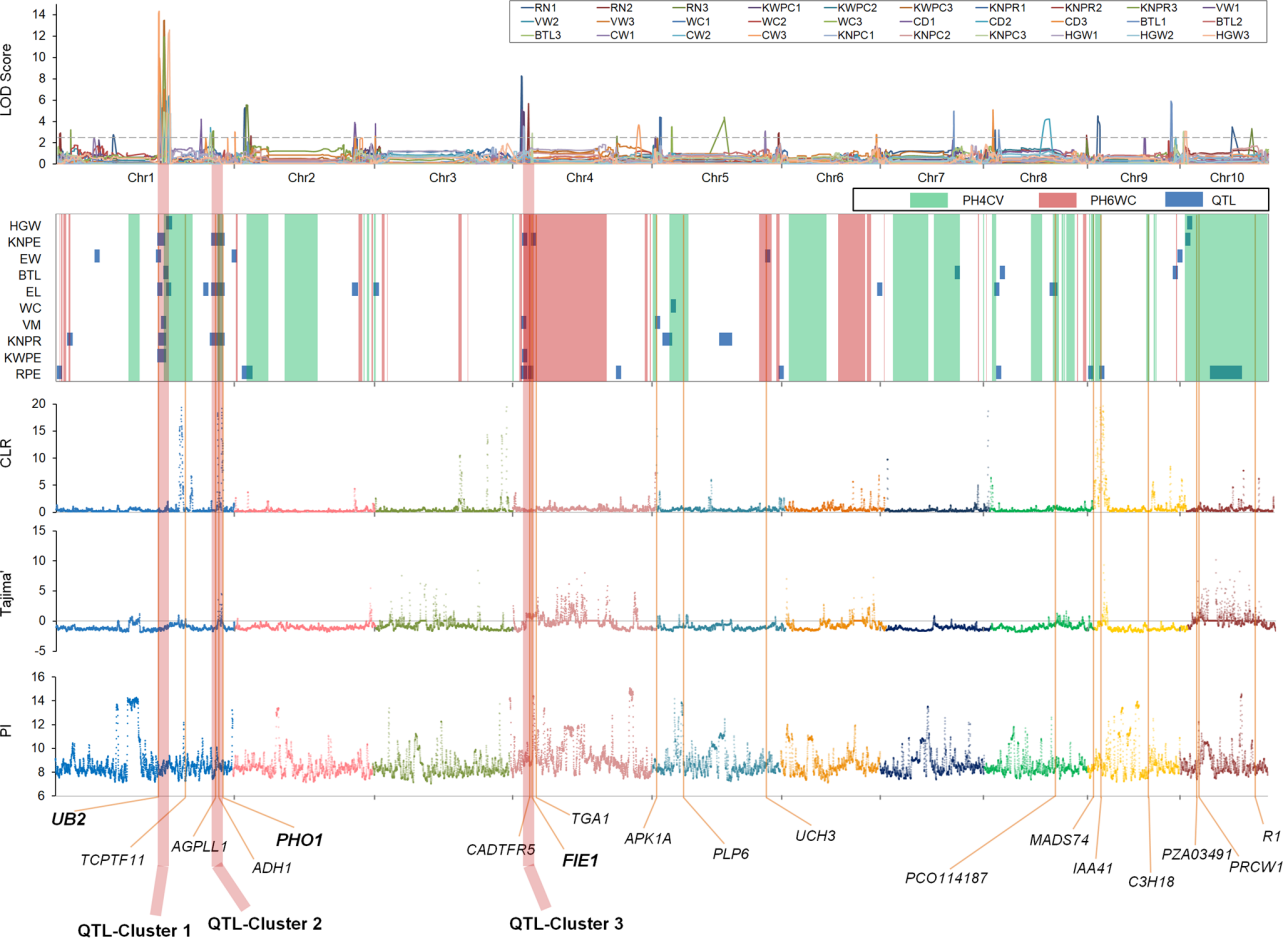
Overview of the IBD conserved regions, selective sweep signals, and LOD scores of quantitative trait loci associated with 10 important yield-related traits.

To examine how selection during breeding has shaped the genomes of the X lines, we performed a genome-wide selection scan of these lines. Sliding windows were used to scan genomic regions based on π, Tajima’s D, and CLR to identify potential selection-related signals. Using the top 10% of values as the threshold, we identified 103, 89, and 131 candidate regions with selective sweeps (SSWs) based on π, Tajima’s D, and CLR, respectively (**Figure 4**). The mean size of individual selected regions for the three methods ranged from 2.3 to 3.7 megabases (Mb). These SSWs were bigger than previously detected SSWs for genetic improvement and domestication, indicating that the genomic regions selected during pedigree breeding are distinct from those selected during long-term genetic improvement.

Interestingly, we identified many selection-related signals concentrated in ICRs. Overall, 96.13% of the ICRs comprising 824.0 Mb overlapped the selection-related signals, including 99.19% of the ICRs from PH4CV and 89.23% of the ICRs from PH6WC. These overlapping regions were probably under artificial selection during the genetic improvement of X lines via breeding.

### 3.4 Important yield-related genes in ICRs and SSWs

Yield-related traits are complex quantitative traits, and the underlying genes are rarely identified via a single strategy. In order to comprehensively identify yield-related genes in ICRs and SSWs, we conducted an initial analysis using multiple annotation methods. Therefore, we used a trait ontology (TO) system for the functional annotation of genes. This system curated all of the available trait-associated site (TAS) information from almost all association mapping studies involving diverse genetic backgrounds (Pan et al., 2019). The TO system, which was curated from TAS information, enabled the functional annotation of genes in ICRs and SSWs. We also combined the data from a previous QTL meta-analysis (Zhou et al., 2020) with the data from the Chinese Rice Data Center (http://www.ricedata.cn/gene/) to determine the annotations of orthologous genes. By integrating multiple annotation methods, we identified 171 genes that control yield-related traits in ICRs and SSWs (**Supplementary Table 3**). For instance, the *MN1* gene (Cheng et al., 1996), which is associated with seed yield, is crucial for the development of the maize endosperm and pedicel. Some studies have confirmed that the *INCW5* gene, which belongs to the same family as *MN1* and encodes a cell wall invertase, is functionally related to *MN1* and also influences seed yield (Juarez-Colunga et al., 2018). The *TGA1* gene, which encodes a protein that functions with TB1 to regulate axillary bud dormancy and apical dominance, has been used to improve crop yield (Dong et al., 2019). Many other genes, including *UB2*, *CADTFR5*, *INCW5*, *CKI7*, *MPK7*, and *FIE2* (Danilevskaya et al., 2003), have also been annotated in ICRs and SSWs. The variety of yield-related genes indicates that they do not affect yield through a single pathway. Instead, yield-related genes affect diverse molecular and physiological pathways and processes during various developmental stages to influence ear formation and maize yield (Zhou, 2020). These genes modulate the yield-related traits of maize cultivars and may be important for the genetic improvement of yield.

To identify the genetic factors controlling yield improvement, we assessed multiple agronomic and yield-related characteristics of a DH population derived from a cross between PH4CV and PH6WC. Through QTL mapping experiments (**Supplementary Figure 3**; **Supplementary Table 4**), we identified multiple QTLs controlling 10 yield-related traits. A total of 55 yield-related QTLs were detected in three environments, distributed among the 10 maize chromosomes. The number of QTLs per trait varied from 1 (for WC) to 13 (for RPE), and the phenotypic variance explained (PVE) by each QTL ranged from 3.17% to 22.06%. Among these QTLs were stable QTLs detected in at least two environments (e.g., *qknpr1-2*, *qvw1*, *qel1-1*, *qew1-2*, and *qknpe1-1*) with LOD scores ranging from 3.42 to 14.31 and PVE values between 6.48% and 19.27%. Notably, most QTLs controlling yield-related traits overlapped with the SSWs and ICRs. Specifically, 69.09% and 58.18% of the QTLs were located in or near SSWs and ICRs, respectively. These results suggest that maize yield-related QTLs may be enriched and retained in ICRs and SSWs.

The QTLs responsible for yield-related traits were not evenly distributed across the genome. Instead, they were concentrated in three main genomic regions, namely QTL-Cluster 1 (174.3–194.4 Mb on chromosome 1), QTL-Cluster 2 (252.9–285.8 Mb on chromosome 1), and QTL-Cluster 3 (18.7–37.2 Mb on chromosome 4). QTL-Cluster 1 contained 13 QTLs with additive effects for yield-related traits, QTL-Cluster 2 contained 4, and QTL-Cluster 3 contained 7. Furthermore, QTL-Cluster 1 included QTLs controlling KWPE, KNPE, VW, EL, BTL, EW, KNPR, and HGW, whereas QTL-Cluster 2 included QTLs controlling KNPE, EL, and KNPR, and QTL-Cluster 3 included QTLs controlling RPE, KWPE, VW, and KNPE. All three QTL clusters overlapped with both the IBD conserved regions and the selective sweeps regions. For example, QTL-Cluster 2 had the highest CLR and Tajima’s D values (34.32 and −1.149). Both QTL-Clusters 1 and 2 resided in the ICRs from PH4CV, whereas QTL-Cluster 3 was located in ICRs from PH6WC.

By combining multiple genetic evidences, including the selective signals, the IBD conserved regions, and QTL results, we found some genes with strong selection signals, which indicated these genes were more important during breeding process. Finally, three key genes with strongest selection signals were identified in each QTL cluster, respectively. Located in QTL-Cluster 1, the *UB2* gene (Chuck et al., 2014) regulates the development of the spikelet pair meristem and the spikelet meristems, thereby influencing ear morphology and yield (Chuck et al., 2014; Liu et al., 2015). The *PHO1* gene (Grimaud et al., 2008; Satoh et al., 2008; Hwang et al., 2016), located in QTL-Cluster 2, plays a crucial role in the initiation of starch synthesis and the maturation of starch granules in seeds. It encodes a plastidic alpha-glucan phosphorylase and is an ortholog of the rice *OsPho1* (Hwang et al., 2016). QTL-Cluster 3 contains the *FIE1* gene (Springer et al., 2002; Hermon et al., 2007), which is an ortholog of the rice fertilization-independent endosperm gene *OsFIE2* (Na et al., 2012). It plays a critical role in plant growth and seed development. These three key genes are crucial for yield improvement in the breeding of X groups.

## 4 Discussion

### 4.1 Maize X lines formed a new Chinese maize heterotic pattern

Maize was imported into China nearly 500 years ago (Li, 2009). The collection and preservation of maize germplasm resources in China can be traced back to the 1950s, and maize breeding was initiated in the 1960s. The breeding history of single-cross hybrids in China has been recorded since the 1970s (Li, 1998; Li, 2009). Some inbred lines, including those from the LAN, HIL, LRC, DR, and P groups, have been important for single-cross hybridizations. For example, Mo17 and its derived lines were widely used for breeding in the 1970s and 1980s, resulting in more than 100 hybrids derived from Mo17 and dozens of hybrids derived from Mo17-related lines. Additionally, HIL and Domestic Reid group lines were first used for breeding in the 1980s and 1990s. Using Huangzaosi or its descendants as parental lines, breeders have developed and released more than 70 inbred lines and 80 important hybrids (Li and Wang, 2010). Hybrids derived from HILs are cultivated on more than 60% of the maize fields in China (Li et al., 2019). Lines belonging to the Domestic Reid group, such as Shen5003, Tie7922, and Ye478, and the related lines have also been widely cultivated (Zeng et al, 1996). The LRC and P groups have contributed to Chinese maize breeding since the 1990s and 2000s. Inbred lines developed from the US hybrid P78599, such as Qi319, P178, and Shen137, as well as the related P group lines have been commonly used for breeding. Breeders using Dan340 (LRC group) or its descendants as parental lines generated and released more than 100 important hybrids.

The US hybrid X1132X has recently been used as the base material for maize breeding involving multiple selection conditions (e.g., high planting densities, large populations, strict selection, and the pyramiding of elite lines from the same heterotic group). Consequently, many elite maize inbred lines, including X lines such as Jing724, Jing725, Jing464, and JingMC01, have been generated. The X lines derived from HIL parents exhibit strong heterosis (Li et al., 2019). The use of X lines as the parents for breeding has resulted in dozens of approved hybrids (**Supplementary Table 1**). Examples include the representative hybrid Jingke968, which was bred using Jing724 as the female parent. Over the last four years, Jingke968 has been cultivated extensively on more than 20 million mu (1.3 × 10^6^ hm^2^) (Zhao et al., 2020). Another excellent hybrid, Jingnongke728, which was bred using JingMC01 as the female parent, has been widely cultivated in the Huang-Huai-Hai region because it is suitable for mechanical grain harvesting (Zhao et al., 2020).

We developed a maize panel comprising 512 inbred lines, which were divided into eight subgroups (HIL, LRC, DR, P, X, IDT, LAN, and Reid). The phylogenetic tree and PCA data revealed a consistent relationship among population structures. The HIL, LRC, DR, P, LAN, IDT, and Reid subgroups were identified in previous studies (Li et al., 2005; Wang et al, 2008b; Lu et al., 2009; Yan et al., 2009; Liu et al, 2012; Wu et al, 2014; Zhang et al, 2018). Because X lines were selected and bred recently (Zhao et al., 2017; Zhao et al., 2018), they have not been studied in detail and the genome-wide changes during pedigree breeding remain unknown. In the current study, we revealed clear genetic relationships among these eight subgroups. More specifically, HIL and LRC (H&L) are distantly related to the other subgroups (**Figures 1A, C** **)**. This result was consistent with the primary heterotic pattern non-H&L × H&L (i.e., introduced germplasm × local germplasm), which produced most of the important hybrids (Zeng, 1990; Wang et al., 1997; Wu et al., 2014). On the basis of the clear difference between the X group and the other groups, including other non-H&L groups (**Figures 1B, C** **)**, X × HIL was identified as a new heterotic pattern in China.

### 4.2 The ICRs and SSWs are important functional genomic blocks

Genetic improvements resulting from breeding have left genomic footprints. A pedigree analysis can reveal the genomic changes occurring during maize breeding. The ICRs and SSWs provide insights into artificially selected genomic regions. Elucidating the functions of these regions is a prerequisite for applying them to genetically improve X lines through breeding. Many previous studies identified the important functional genomic blocks. Wu et al. (2014) detected 15 conserved regions derived from Huangzaosi, which suggested that these conserved IBD regions are associated with important functions. Zhou et al (2016) proved that 26.22% of the Huanghuazhan genome comprises strictly conserved key IBD regions that have frequently been selected in pedigree breeding systems. Li et al. (2019) detected 719 selective sweeps, among which 437 (60.53%) regions overlapped with the ICR and were enriched with yield-related genes/QTLs. In a recent investigation, Wang et al. (2020) identified 160 loci underlying adaptive agronomic phenotypes and more than 1,800 important genomic regions with genes related to traits selected for in modern breeding programs. All of these studies confirmed that ICRs and SSWs are important functional genomic blocks.

In this study, we identified 103, 89, and 131 SSWs based on π, Tajima’s D, and CLR values, respectively. We also detected 29 and 28 ICRs from the parents PH4CV and PH6WC, respectively. Integrating multiple annotation methods, including TO functional annotations, QTL meta-analyses, and homologous gene functional annotations, indicated many yield-related genes exist in ICRs and SSWs. Of these genes, 113 were in the ICR and SSW overlapping regions in PH4CV, whereas 58 were in the ICR and SSW overlapping regions in PH6WC. For example, *UB2* (Chuck et al., 2014), *INCW5* (Wang et al., 2008a), *CKI7* (Garza-Aguilar et al, 2019), *MPK7* (Trevisan et al., 2019), *FIE2*, and *MN1* (Cheng et al., 1996) were detected in both ICRs and SSWs in PH4CV, whereas *TGA1* (Wang et al., 2005; Dong et al., 2019), *FIE1*, and *CADTFR5* (Zhou et al., 2020) were detected in both ICRs and SSWs in PH6WC. The results presented here provide a valuable resource for mining of superior alleles for yield improvement. Furthermore, our QTL mapping confirmed above results and further indicated the key genes in the breeding improvement in X group.

### 4.3 Pedigree analyses revealed the characteristics of artificial selection

Meiotic recombination is a crucial driving force for enhancing crop genetic diversity during breeding, and thus, for improving traits (Zhang and Gaut, 2003; Meunier and Duret, 2004; Gaut et al., 2007; Li et al., 2007a; Li et al., 2007b; Kulathinal et al., 2008; Kent et al., 2012; Pan et al., 2016). Pedigree breeding, which involves lines derived from the F_1_ generation, results in substantial genomic recombinations and the pyramiding of favorable alleles from the parents into the inbred lines.

In this study, we confirmed the pyramiding of PH4CV and PH6WC genes associated with important traits in the X lines (**Figure 2**). We also observed significant improvements in maize yield-related traits, including RPE, KNPE, and KWPE, in almost all X lines compared to the corresponding traits in the parents. Our hypothesis is that the X lines retained favorable alleles from the two parents and this was confirmed by a QTL analysis. For instance, an analysis of the cumulative additive effect (CAE) of the QTLs for RPE indicated that the QTLs had a greater CAE in most of the X lines, such as Jing72464, Jing724, and JingMC01, than in the parents (**Supplementary Tables 5, 6**). This suggests that gene pyramiding has been successfully applied during the pedigree breeding of X lines. Moreover, yield-related QTLs were enriched and retained in ICRs and SSWs, implying that ICRs and SSWs are important functional genomic blocks inherited from the parents, which suggests that the X lines retained these favorable genomic regions. The functional characterization of the genes in these important functional blocks, which were localized to overlapping ICRs and SSWs, indicates that the parents differentially contributed to the X line phenotypes (**Supplementary Figure 4**). Specifically, PH4CV contributed more yield-related genes, whereas PH6WC contributed more genes related to plant growth and development. We found more yield-related QTLs and candidate genes (113/171) in or near the ICRs of PH4CV than in or near the ICRs of PH6WC, which is consistent with the functional annotations (**Figure 4**). This finding is also consistent with the opinions of breeders.

## Data availability statement

The data presented in this study are deposited in the NCBI Sequence Read Archive repository, accession number PRJNA974168.

## Author contributions

JZ, WS and YW designed the experiments. ZL and CL analyzed the data and wrote the manuscript. RZ, MD, JW, AS, ShuaiW, XS, YZhao, ShuaisW, and YZhan performed to the field work and constructed population. HT, HY, LX, FW, ZS, and XW took part in the part of experiments and the manuscript modification. All authors contributed to the article and approved the submitted version.
