# Supplementary material for: Genomic analysis of a new heterotic maize group reveals key loci for pedigree breeding
Source: Front Plant Sci. 2023 Aug 11;14:1213675. doi: 10.3389/fpls.2023.1213675 (PMC10451083; doi:10.3389/fpls.2023.1213675)
Supplement: Supplementary file 1 [file DataSheet_1.docx]

Supplementary Material

# Supplementary Tables

Supplementary Table 1. Representative approved cultivars with X lines as parents

| **No．** | **Inbred line** | **Cultivars** | **Approved number** | **Parents** |
| --- | --- | --- | --- | --- |
| 1 | Jing724 | Jingke968 | Guoshenyu2011007 | Jing724 × Jing92 |
|  |  | JK9681 | Guoshenyu20180231 | Jing724 × Jing92H |
|  |  | MC738 | Guoshenyu20190033 | Jing724 × Jing2416 |
| 2 | Jing725 | Jingke665 | Guoshenyu20190028 | Jing725 × Jing92 |
|  |  | Fengle303 | Guoshenyu20176055 | Jing725 × Jing2416 |
| 3 | Jing464 | NK718 | Guoshenyu20180262 | Jing464 × Jing2416 |
| 4 | JingMC01 | JK9685 | Guoshenyu20190104 | JingMC01 × Jing92H |
|  |  | Jingnongke729 | Guoshenyu20190105 | JingMC01 × Jing2418 |
|  |  | Jingnongke728 | Guoshenyu20170007 | JingMC01 × Jing2416 |
| 5 | JingB547 | Shuoqiu518 | Guoshenyu20186114 | JingB547 × Jing2416D |
|  |  | MC812 | Guoshenyu20190284 | JingB547 × Jing2416 |
| 6 | DH382 | Denghai605 | Guoshenyu2010009 | DH351×DH382 |
|  |  | Denghai662 | Guoshenyu2009010 | DH371×DH382 |
|  |  | Denghai685 | Guoshenyu2015011 | DH382×DH357-14 |
| 7 | 91277 | Shandan609 | Guoshenyu2016001 | 91227 × Chang7-2 |
| 8 | YuA9241 | YuDan606 | Guoshenyu2015018 | YuA9241 × XinA3 |
| 9 | Jing72464 | MC121 | Guoshenyu20180070 | Jing72464 × Jing2416 |
| 10 | JingDH3345 | SK4517 | Guoshenyu20190263 | JingDH3345 × Jing2417 |
| 11 | Jing88 | Jingnongke828 | Jingshenyu20170002 | Jing88 × Jing2416 |
| 12 | Jing4055 | MC4592 | Jingshenyu2014001 | Jing4055 × Jing92 |
| 13 | X24621 | Nongda372 | Guoshenyu2015014 | X24621 × BA702 |

Supplementary Table 2 Resequencing of elite X lines and their parents

| **Name** | **Category** | **Reads (M)** | **Bases (G)** | **Map reads （%）** | **Map reads** | **Depth (X)** | **Coverage (%)** |
| --- | --- | --- | --- | --- | --- | --- | --- |
| PH4CV | Parents | 355.64 | 47.86 | 89.80 | 314123712 | 23.24 | 88.90 |
| PH6WC | Parents | 474.86 | 64.67 | 91.52 | 429212362 | 31.40 | 91.32 |
| DH382 | X lines | 155.61 | 22.73 | 88.87 | 136266112 | 11.04 | 88.80 |
| Jing724 | X lines | 183.13 | 26.86 | 88.66 | 159907952 | 13.04 | 87.15 |
| Jing725 | X lines | 393.95 | 53.81 | 90.01 | 349216966 | 26.13 | 89.36 |
| Jing464 | X lines | 350.51 | 48.59 | 89.99 | 310638312 | 23.60 | 89.47 |
| JingMC01 | X lines | 458.36 | 61.74 | 90.88 | 410270768 | 29.98 | 89.91 |
| D9H | X lines | 371.83 | 50.03 | 90.99 | 333627112 | 24.29 | 89.76 |
| B547 | X lines | 376.69 | 51.43 | 90.13 | 334492584 | 24.97 | 89.47 |
| 91277 | X lines | 157.12 | 23.57 | 90.62 | 140557860 | 11.44 | 88.04 |
| YuA9241 | X lines | 143.86 | 21.58 | 89.37 | 126596320 | 10.48 | 86.26 |
| Jing72464 | X lines | 153.65 | 23.05 | 89.03 | 134655884 | 11.19 | 86.98 |
| JingDH3345 | X lines | 148.83 | 22.32 | 88.88 | 130288166 | 10.84 | 86.70 |
| Jing88 | X lines | 149.51 | 22.43 | 89.46 | 131775412 | 10.89 | 86.65 |
| Jing4055 | X lines | 208.11 | 31.22 | 90.61 | 185744658 | 15.16 | 87.70 |
| X24621 | X lines | 153.36 | 23.00 | 89.57 | 135414266 | 11.17 | 87.14 |
| D9B | X lines | 83.17 | 12.48 | 93.50 | 76636572 | 6.06 | 81.47 |

Supplementary Table 3. The list of selective target genes with function reported.

| **Gene Model ID** | **Chr** | **Start** | **End** | **Rice Homologous Gene** | **Arabidopsis Homologous Gene** | **Gene Symbol** | **Cite** | **Full Name** | **Notes** |
| --- | --- | --- | --- | --- | --- | --- | --- | --- | --- |
| *GRMZM2G166687* | 1 | 214,354,391 | 214,355,375 | *LOC_Os08g33530* | *AT3G18550.2* | *TCPTF11* |  | TCP-transcription factor 11 | TCP family transcription factor, putative, expressed |
| *GRMZM2G391936* | 1 | 273,514,151 | 273,518,985 | *LOC_Os03g52460* | *AT5G19220.1* | *AGPLL1* |  | ADP glucose pyrophosphorylase large subunit leaf1AGPL4 | glucose-1-phosphate adenylyltransferase large subunit, putative, expressed |
| *GRMZM2G442658* | 1 | 274,050,254 | 274,054,148 | *LOC_Os11g10480* | *AT1G77120.1* | *ADH1* |  | alcohol dehydrogenase1 | dehydrogenase, putative, expressed |
| *GRMZM2G047855* | 1 | 279,484,878 | 279,490,883 | *LOC_Os07g02350* | *AT5G67380.1* | *CKA2* |  | CK2 protein kinase alpha 2 | casein kinase II subunit alpha-2, putative, expressed |
| *GRMZM5G814279* | 9 | 7,111,132 | 7,121,722 | *LOC_Os02g52340* | *AT2G22540.1* | *MADS74* |  | MADS-transcription factor 74 | OsMADS22 - MADS-box family gene with MIKCc type-box, expressed |
| *GRMZM2G109720* | 9 | 13,496,885 | 13,502,062 | *LOC_Os06g08390* | *AT5G58200.2* | *CSU471* |  |  | Ser/Thr protein phosphatase family protein, putative, expressed |
| *GRMZM2G014240* | 9 | 14,328,479 | 14,333,699 | *LOC_Os06g08080* | *AT1G15690.1* | *AY103622* |  |  | inorganic H+ pyrophosphatase, putative, expressed |
| *GRMZM2G001799* | 9 | 16,255,609 | 16,257,497 | *LOC_Os02g56120* | *AT4G14550.1* | *IAA41* |  | Aux/IAA-transcription factor 41 | OsIAA9 - Auxin-responsive Aux/IAA gene family member, expressed |
| *GRMZM2G151689* | 9 | 101,513,894 | 101,516,776 | *LOC_Os06g41384* | *AT5G06770.1* | *C3H18* |  | C3H-transcription factor 318 | zinc finger C-x8-C-x5-C-x3-H type family protein, expressed |
| *GRMZM2G472703* | 10 | 36,254,235 | 36,258,217 | *LOC_Os01g12390* | *AT4G23740.1* | *PZA03491* |  |  | inactive receptor kinase At2g26730 precursor, putative, expressed |
| *GRMZM2G049915* | 10 | 36,785,988 | 36,787,819 |  |  | *PRCW1* |  | proline rich cell wall protein1 |  |
| *GRMZM2G118063* | 10 | 58,000,726 | 58,009,266 | *LOC_Os08g08820* | *AT4G04890.1* | *HB17* |  | Homeobox-transcription factor 17 | homeobox and START domains containing protein, putative, expressed |
| *GRMZM2G041963* | 10 | 66,889,695 | 66,890,958 | *LOC_Os08g13000* | *AT3G06190.1* | *MAB28* |  | math-btb28 | MBTB20 - Bric-a-Brac, Tramtrack, Broad Complex BTB domain with Meprin and TRAF Homology MATH domain, expressed |
| *GRMZM2G112782* | 10 | 71,063,036 | 71,066,861 | *LOC_Os08g14640* | *AT4G30240.1* | *PZA01677* |  |  | syntaxin 6, N-terminal domain containing protein, expressed |
| *AC185587.3_FG003* | 1 | 203,132,949 | 203,134,532 | *LOC_Os03g03790* | *AT1G65880.1* |  |  |  | AMP-binding domain containing protein, expressed |
| *GRMZM2G154124* | 1 | 206,200,059 | 206,204,623 | *LOC_Os08g44020* | *AT1G09890.1* |  |  |  | rhamnogalacturonate lyase, putative, expressed |
| *GRMZM2G160917* | 1 | 188,215,376 | 188,219,483 | *LOC_Os08g39890* | *AT2G42200.1* | *UB2* | Chuck et al., 2014;  Liu et al., 2015 | unbranched2 | OsSPL14 - SBP-box gene family member, expressed |
| *GRMZM2G028726* | 1 | 208,478,252 | 208,483,075 | *LOC_Os09g26999* |  | *BNLG1556* |  |  | keratin-associated protein 5-4, putative, expressed |
| *GRMZM2G036455* | 1 | 227,569,042 | 227,572,130 | *LOC_Os10g35370* | *AT5G54190.1* |  |  |  | oxidoreductase, short chain dehydrogenase/reductase family domain containing family, expressed |
| *GRMZM2G087095* | 1 | 277,377,468 | 277,415,566 | *LOC_Os03g54170* | *AT3G02310.1* |  |  |  | OsMADS34 - MADS-box family gene with MIKCc type-box, expressed |
| *GRMZM5G826714* | 1 | 277,446,649 | 277,450,112 | *LOC_Os05g32110* | *AT5G60920.1* |  |  |  | COBRA, putative, expressed |
| *GRMZM2G074158* | 1 | 278,250,612 | 278,259,617 | *LOC_Os03g55090* | *AT3G29320.1* | *PHO1* | Grimaud et al. 2008;  Hwang et al., 2016;  Satoh et al., 2008 | starch phosphorylase1 | alpha-glucan phosphorylast isozyme, putative, expressed |
| *GRMZM2G465188* | 2 | 22,496,748 | 22,498,854 | *LOC_Os05g32110* | *AT3G02210.1* |  |  |  | COBRA, putative, expressed |
| *GRMZM2G119689* | 2 | 57,435,843 | 57,439,124 | *LOC_Os04g33740* | *AT3G52600.1* | *MN1* | Cheng et al., 1996 | miniature seed1 | glycosyl hydrolases, putative, expressed |
| *GRMZM2G138676* | 5 | 1,357,389 | 1,363,381 | *LOC_Os03g62500* | *AT1G09840.1* | *GPM645* |  |  | CGMC_GSK.5 - CGMC includes CDA, MAPK, GSK3, and CLKC kinases, expressed |
| *GRMZM2G144042* | 5 | 2,284,054 | 2,290,332 | *LOC_Os03g60710* | *AT1G07570.1* | *APK1A* |  |  | protein kinase domain containing protein, expressed |
| *GRMZM2G089448* | 5 | 4,918,380 | 4,920,036 | *LOC_Os03g57240* | *AT5G48890.1* |  |  |  | ZOS3-19 - C2H2 zinc finger protein, expressed |
| *GRMZM2G427729* | 5 | 42,893,993 | 42,896,091 | *LOC_Os06g46350* | *AT4G29800.1* | *PLP6* |  |  | PLA IIIA/PLP7, putative, expressed |
| *AC207656.3_FG002* | 5 | 49,282,509 | 49,284,911 | *LOC_Os06g47150* | *AT4G30080.1* | *ARFTF19* |  | ARF-transcription factor 19 | auxin response factor 18, putative, expressed |
| *GRMZM2G393272* | 6 | 74,758,342 | 74,759,985 | *LOC_Os06g06050* | *AT2G42620.1* |  |  |  | OsFBL27 - F-box domain and LRR containing protein, expressed |
| *GRMZM2G002100* | 6 | 74,762,186 | 74,767,526 | *LOC_Os06g06090* | *AT2G43790.1* | *MPK7* | Trevisan et al., 2019 | MAP kinase7 | CGMC_MAPKCMGC_2_ERK.12 - CGMC includes CDA, MAPK, GSK3, and CLKC kinases, expressed |
| *GRMZM2G151536* | 8 | 96,440,916 | 96,444,449 | *LOC_Os05g37690* | *AT2G39940.1* |  |  |  | OsFBL23 - F-box domain and LRR containing protein, expressed |
| *GRMZM2G071986* | 9 | 5,319,666 | 5,328,954 | *LOC_Os12g17080* | *AT1G34160.1* |  |  |  | pentatricopeptide, putative, expressed |
| *GRMZM2G136032* | 9 | 6,467,685 | 6,469,385 | *LOC_Os02g44630* | *AT4G00430.1* | *PIP1F* |  | plasma membrane intrinsic protein1 | aquaporin protein, putative, expressed |
| *GRMZM2G343769* | 9 | 7,870,479 | 7,871,904 | *LOC_Os02g52480* | *AT5G48820.2* | *CKI7* | Garza-Aguilar et al., 2018 | cyclin-dependent kinase inhibitor7 | cyclin-dependent kinase inhibitor, putative, expressed |
| *GRMZM2G071249* | 9 | 17,669,887 | 17,679,026 | *LOC_Os06g06530* | *AT5G53330.1* |  |  |  | proline-rich cell wall protein-like, putative, expressed |
| *GRMZM2G020216* | 9 | 18,879,527 | 18,885,415 | *LOC_Os06g06090* | *AT2G43790.1* | *MPK2* |  | MAP kinase2 | CGMC_MAPKCMGC_2_ERK.12 - CGMC includes CDA, MAPK, GSK3, and CLKC kinases, expressed |
| *GRMZM2G405203* | 9 | 18,894,557 | 18,897,788 | *LOC_Os06g06050* | *AT2G42620.1* |  |  |  | OsFBL27 - F-box domain and LRR containing protein, expressed |
| *GRMZM5G883993* | 9 | 100,869,848 | 100,876,008 | *LOC_Os06g41730* | *AT2G17410.1* | *ARID10* |  | ARID-transcription factor 10 | ARID/BRIGHT DNA-binding domain-containing protein, putative, expressed |
| *GRMZM2G114680* | 9 | 103,353,117 | 103,362,197 | *LOC_Os06g40780* | *AT1G55580.1* | *GRAS70* |  | GRAS-transcription factor 70 | MONOCULM 1, putative, expressed |
| *GRMZM2G125513* | 9 | 114,942,808 | 114,947,032 | *LOC_Os10g41310* | *AT3G60320.1* |  |  |  | DUF630/DUF632 domains containing protein, putative, expressed |
| *GRMZM2G073860* | 10 | 27,467,514 | 27,470,619 | *LOC_Os12g44020* | *AT2G16430.2* |  |  |  | Ser/Thr protein phosphatase family protein, putative, expressed |
| *GRMZM2G155729* | 10 | 63,796,931 | 63,801,064 | *LOC_Os08g10600* | *AT1G31070.2* |  |  |  | UTP--glucose-1-phosphate uridylyltransferase, putative, expressed |
| *GRMZM2G020054* | 10 | 65,437,299 | 65,438,594 | *LOC_Os04g52090* | *AT3G20310.1* | *EREB54* |  | AP2-EREBP-transcription factor 54 | AP2 domain containing protein, expressed |
| *GRMZM2G471335* | 10 | 78,559,710 | 78,564,240 | *LOC_Os11g04954* | *AT3G22880.1* |  |  |  | DNA repair protein Rad51, putative, expressed |
| *GRMZM2G148924* | 10 | 84,106,322 | 84,110,776 | *LOC_Os08g04270* | *AT3G20740.1* | *FIE2* | Danilevskaya et al., 2003 | fertilization independent endosperm2 | WD domain, G-beta repeat domain containing protein, expressed |
| *GRMZM2G062541* | 10 | 87,704,026 | 87,706,060 | *LOC_Os05g04740* | *AT1G09530.1* | *BHLH117* |  | bHLH-transcription factor 117 | helix-loop-helix DNA-binding domain containing protein, expressed |
| *GRMZM2G357737* | 10 | 89,082,654 | 89,087,874 | *LOC_Os01g12890* | *AT5G11530.1* |  |  |  | expressed protein |
| *AC199571.3_FG003* | 10 | 93,238,153 | 93,239,781 | *LOC_Os01g12890* |  |  |  |  | expressed protein |
| *GRMZM2G420801* | 10 | 101,173,508 | 101,177,424 | *LOC_Os04g08740* | *AT3G04580.1* | *ETR2* |  | ethylene receptor homolog2 | ethylene receptor, putative, expressed |
| *GRMZM2G095725* | 10 | 114,256,599 | 114,259,719 | *LOC_Os04g33740* | *AT3G52600.1* | *INCW5* | Wang et al., 2008a | invertase cell wall5 | glycosyl hydrolases, putative, expressed |
| *GRMZM5G870959* | 10 | 121,772,419 | 121,777,860 | *LOC_Os04g38480* | *AT1G71830.1* | *SERK1* |  | somatic embryogenesis receptor-like kinase1 | BRASSINOSTEROID INSENSITIVE 1-associated receptor kinase 1 precursor, putative, expressed |
| *GRMZM2G135470* | 10 | 123,393,683 | 123,398,962 | *LOC_Os04g39020* | *AT3G48170.1* |  |  |  | aldehyde dehydrogenase, putative, expressed |
| *GRMZM2G028622* | 10 | 146,494,874 | 146,496,217 | *LOC_Os04g56780* | *AT2G17950.1* | *HB122* |  | Homeobox-transcription factor 122 | homeobox domain containing protein, expressed |
| *GRMZM2G303157* | 1 | 207,171,162 | 207,176,414 | *LOC_Os08g36490* | *AT3G54630.1* |  |  |  | kinetochore protein, putative, expressed |
| *GRMZM2G303149* | 1 | 207,177,188 | 207,178,075 | *LOC_Os08g43490* | *AT2G17880.1* |  |  |  | heat shock protein DnaJ, putative, expressed |
| *GRMZM2G428554* | 1 | 210,641,732 | 210,645,615 | *LOC_Os08g34640* | *AT1G73080.1* |  |  |  | receptor-like protein kinase precursor, putative, expressed |
| *GRMZM2G390041* | 1 | 214,346,308 | 214,348,412 | *LOC_Os11g42540* |  |  |  |  | oxidoreductase, aldo/keto reductase family protein, putative, expressed |
| *GRMZM2G079428* | 1 | 229,910,587 | 229,926,888 | *LOC_Os10g34400* | *AT4G33630.1* |  |  |  | expressed protein |
| *GRMZM2G090043* | 1 | 273,511,950 | 273,512,701 | *LOC_Os03g52370* |  |  |  |  | PIII4 - Proteinase inhibitor II family protein precursor, expressed |
| *GRMZM2G147900* | 1 | 273,759,019 | 273,760,563 | *LOC_Os03g52620* | *AT1G77405.1* |  |  |  | expressed protein |
| *GRMZM2G131525* | 1 | 273,842,906 | 273,849,374 | *LOC_Os03g52650* | *AT1G08560.1* |  |  |  | syntaxin-related protein, putative, expressed |
| *GRMZM2G131575* | 1 | 273,852,128 | 273,853,789 | *LOC_Os03g52660* | *AT4G09650.1* |  |  |  | ATP synthase F1, delta subunit family protein, putative, expressed |
| *GRMZM2G169671* | 1 | 275,290,500 | 275,294,312 | *LOC_Os03g53270* | *AT5G43830.1* |  |  |  | stem-specific protein TSJT1, putative, expressed |
| *GRMZM2G145950* | 1 | 276,600,412 | 276,602,149 | *LOC_Os03g56700* | *AT3G12870.1* |  |  |  | expressed protein |
| *GRMZM2G446999* | 1 | 276,600,414 | 276,601,032 | *LOC_Os03g56782* | *AT1G31335.1* |  |  |  | expressed protein |
| *GRMZM2G145974* | 1 | 276,603,845 | 276,605,054 | *LOC_Os03g53900* | *AT3G11930.4* |  |  |  | universal stress protein domain containing protein, putative, expressed |
| *GRMZM2G146000* | 1 | 276,605,739 | 276,608,726 | *LOC_Os03g53910* | *AT1G04130.1* |  |  |  | tetratricopeptide repeat domain containing protein, expressed |
| *GRMZM2G123700* | 1 | 277,903,101 | 277,904,720 |  |  |  |  |  |  |
| *GRMZM2G123714* | 1 | 277,910,574 | 277,913,398 | *LOC_Os03g54910* | *AT1G18650.1* |  |  |  | X8 domain containing protein, expressed |
| *GRMZM2G123732* | 1 | 277,915,990 | 277,919,545 | *LOC_Os03g54920* | *AT3G05010.1* |  |  |  | expressed protein |
| *GRMZM2G348578* | 1 | 279,479,453 | 279,483,540 | *LOC_Os03g55380* | *AT4G33910.1* |  |  |  | oxidoreductase, putative, expressed |
| *GRMZM2G047759* | 1 | 279,541,708 | 279,543,303 | *LOC_Os03g55400* | *AT5G67390.1* |  |  |  | expressed protein |
| *GRMZM2G348563* | 1 | 279,544,172 | 279,544,977 |  |  |  |  |  |  |
| *GRMZM2G107689* | 1 | 280,559,358 | 280,566,360 | *LOC_Os03g55620* | *AT2G45490.1* |  |  |  | CAMK_CAMK_like_Aur_like.2 - CAMK includes calcium/calmodulin depedent protein kinases, expressed |
| *GRMZM5G800014* | 2 | 56,111,774 | 56,113,119 | *LOC_Os04g33990* | *AT3G44220.1* |  |  |  | harpin-induced protein 1 domain containing protein, expressed |
| *GRMZM2G001653* | 2 | 56,736,412 | 56,737,906 | *LOC_Os04g33830* | *AT1G08380.1* |  |  |  | membrane protein, putative, expressed |
| *GRMZM2G056996* | 2 | 58,003,495 | 58,033,733 | *LOC_Os12g36620* | *AT4G13650.1* |  |  |  | pentatricopeptide, putative, expressed |
| *GRMZM5G875516* | 2 | 229,679,539 | 229,679,846 |  |  |  |  |  |  |
| *GRMZM2G078013* | 2 | 229,691,128 | 229,697,078 | *LOC_Os11g39320* | *AT3G46730.1* |  |  |  | LZ-NBS-LRR class, putative, expressed |
| *GRMZM2G041549* | 2 | 229,800,854 | 229,802,637 | *LOC_Os11g39640* | *AT4G33565.1* |  |  |  | zinc finger, C3HC4 type domain containing protein, expressed |
| *GRMZM2G041642* | 2 | 229,802,703 | 229,814,523 | *LOC_Os11g39650* |  |  |  |  | WD domain, G-beta repeat domain containing protein, expressed |
| *GRMZM2G339848* | 4 | 3,759,509 | 3,766,867 | *LOC_Os05g09630* | *AT4G03090.1* |  |  |  | homeobox domain containing protein, expressed |
| *GRMZM5G845021* | 4 | 4,740,446 | 4,742,408 | *LOC_Os11g41650* | *AT3G03900.1* |  |  |  | adenylyl-sulfate kinase, putative, expressed |
| *AC204619.3_FG003* | 8 | 146,459,675 | 146,460,067 | *LOC_Os01g45700* |  |  |  |  | expressed protein |
| *GRMZM2G700700* | 8 | 146,461,064 | 146,461,390 |  |  |  |  |  |  |
| *GRMZM5G894582* | 8 | 146,466,455 | 146,467,438 | *LOC_Os01g45700* |  | *PCO114187* |  |  | expressed protein |
| *AC209664.3_FG002* | 9 | 4,651,776 | 4,654,753 | *LOC_Os06g12280* | *AT3G25140.1* |  |  |  | glycosyl transferase 8 domain containing protein, putative, expressed |
| *GRMZM2G102494* | 9 | 4,663,526 | 4,665,097 |  |  |  |  |  |  |
| *GRMZM2G003167* | 9 | 4,777,177 | 4,778,055 | *LOC_Os06g11130* | *AT1G49660.1* |  |  |  | gibberellin receptor GID1L2, putative, expressed |
| *GRMZM2G058560* | 9 | 6,263,844 | 6,268,282 | *LOC_Os06g11640* | *AT4G08960.1* |  |  |  | serine/threonine-protein phosphatase 2A activator 2, putative, expressed |
| *GRMZM2G058549* | 9 | 6,272,227 | 6,272,883 |  |  |  |  |  |  |
| *GRMZM2G135960* | 9 | 6,491,475 | 6,492,651 | *LOC_Os06g11610* | *AT5G51440.1* |  |  |  | heat shock 22 kDa protein, mitochondrial precursor, putative, expressed |
| *GRMZM2G008033* | 9 | 6,573,679 | 6,574,655 | *LOC_Os06g11510* | *AT3G06868.1* |  |  |  | expressed protein |
| *GRMZM2G307440* | 9 | 6,579,401 | 6,581,717 | *LOC_Os06g11135* | *AT1G49640.1* |  |  |  | gibberellin receptor GID1L2, putative, expressed |
| *GRMZM2G174048* | 9 | 7,105,655 | 7,108,037 | *LOC_Os06g11310* | *AT2G15780.1* |  |  |  | plastocyanin-like domain containing protein, putative, expressed |
| *GRMZM2G017244* | 9 | 7,266,420 | 7,268,133 | *LOC_Os06g11304* | *AT4G16835.1* |  |  |  | expressed protein |
| *AC217577.1_FG003* | 9 | 8,005,200 | 8,005,709 |  |  |  |  |  |  |
| *GRMZM2G080696* | 9 | 8,015,075 | 8,016,841 | *LOC_Os06g10980* | *AT2G03220.1* |  |  |  | xyloglucan fucosyltransferase, putative, expressed |
| *GRMZM2G116491* | 9 | 13,154,369 | 13,156,287 | *LOC_Os02g55190* | *AT4G31805.1* |  |  |  | expressed protein |
| *AC212720.3_FG006* | 9 | 13,502,341 | 13,503,017 |  |  |  |  |  |  |
| *GRMZM2G128515* | 9 | 14,318,120 | 14,321,708 | *LOC_Os06g08110* | *AT1G80530.1* |  |  |  | nodulin, putative, expressed |
| *GRMZM2G428020* | 9 | 14,322,329 | 14,323,553 | *LOC_Os06g08100* | *AT1G15760.1* |  |  |  | jp18, putative, expressed |
| *GRMZM5G858599* | 9 | 14,325,590 | 14,326,252 |  |  |  |  |  |  |
| *AC234158.1_FG005* | 9 | 112,508,146 | 112,513,862 | *LOC_Os02g50840* | *AT3G48050.1* |  |  |  | BAH domain containing protein, putative, expressed |
| *GRMZM2G087655* | 10 | 30,198,083 | 30,201,459 | *LOC_Os08g02050* | *AT1G09600.1* |  |  |  | protein kinase family protein, putative, expressed |
| *GRMZM2G087692* | 10 | 30,201,516 | 30,205,190 |  | *AT1G22800.1* |  |  |  |  |
| *GRMZM2G173055* | 10 | 36,259,684 | 36,260,157 |  |  |  |  |  |  |
| *GRMZM2G010406* | 10 | 38,530,684 | 38,542,590 | *LOC_Os12g13320* | *AT4G24830.1* |  |  |  | argininosuccinate synthase, chloroplast precursor, putative, expressed |
| *GRMZM2G155357* | 10 | 66,645,576 | 66,650,999 |  |  |  |  |  |  |
| *GRMZM2G155392* | 10 | 66,651,522 | 66,652,359 | *LOC_Os10g24800* |  |  |  |  | hypothetical protein |
| *GRMZM2G454472* | 10 | 66,654,901 | 66,656,458 | *LOC_Os01g52430* | *AT3G17450.1* |  |  |  | hAT dimerisation domain-containing protein, putative, expressed |
| *AC208564.3_FG004* | 1 | 186,769,873 | 186,771,159 | *LOC_Os03g54170* | *AT2G03710.3* |  |  |  | OsMADS34 - MADS-box family gene with MIKCc type-box, expressed |
| *GRMZM2G051806* | 6 | 130,163,346 | 130,166,258 | *LOC_Os05g09500* | *AT2G19860.1* | *HEX7* |  | hexokinase7 | hexokinase, putative, expressed |
| *AC213884.3_FG002* | 6 | 140,735,955 | 140,737,064 | *LOC_Os05g28320* | *AT4G37260.1* | *MYB119* |  | MYB-transcription factor 119 | myb-like DNA-binding domain containing protein, putative, expressed |
| *GRMZM2G103945* | 6 | 133,537,405 | 133,539,720 |  |  | *TIP4A* |  | tonoplast intrinsic protein4 |  |
| *GRMZM2G171390* | 3 | 146,639,771 | 146,642,645 | *LOC_Os10g41780* | *AT1G44446.3* |  |  |  | chlorophyllide a oxygenase, chloroplast precursor, putative, expressed |
| *GRMZM2G089995* | 4 | 26,450,939 | 26,451,894 | *LOC_Os04g52090* | *AT3G20310.1* | *EREB209* |  | AP2-EREBP-transcription factor 209 | AP2 domain containing protein, expressed |
| *GRMZM2G122717* | 4 | 27,843,728 | 27,845,162 | *LOC_Os03g32580* | *AT5G21090.1* |  |  |  | BRASSINOSTEROID INSENSITIVE 1-associated receptor kinase 1 precursor, putative, expressed |
| *GRMZM2G444073* | 4 | 30,460,409 | 30,461,221 | *LOC_Os08g07740* | *AT4G14540.1* | *CADTFR5* | Zhou et al., 2020 | CCAAT-DR1-transcription factor 5 | histone-like transcription factor and archaeal histone, putative, expressed |
| *GRMZM2G118205* | 4 | 35,790,694 | 35,796,700 | *LOC_Os08g04270* | *AT3G20740.1* | *FIE1* | Hermon et al., 2007;  Springer et al., 2002 | fertilization independent endosperm1 | WD domain, G-beta repeat domain containing protein, expressed |
| *GRMZM2G422137* | 4 | 38,623,038 | 38,627,011 | *LOC_Os03g11734* | *AT1G51340.2* |  |  |  | MATE efflux protein, putative, expressed |
| *GRMZM2G300141* | 4 | 39,807,855 | 39,808,685 | *LOC_Os08g01670* | *AT5G64620.1* |  |  |  | invertase/pectin methylesterase inhibitor family protein, putative, expressed |
| *GRMZM2G164735* | 4 | 43,266,139 | 43,268,012 | *LOC_Os10g02584* | *AT1G14685.1* | *BBR3* |  | BBR/BPC-transcription factor 3 | GAGA-binding protein, putative, expressed |
| *GRMZM2G101511* | 4 | 44,534,815 | 44,539,478 | *LOC_Os08g41940* | *AT5G50670.1* | *TGA1* | Wang et al., 2005;  Dong et al., 2019 | teosinte glume architecture1 | OsSPL16 - SBP-box gene family member, expressed |
| *GRMZM2G107289* | 4 | 47,746,076 | 47,751,492 | *LOC_Os08g42540* |  |  |  |  | ubiquitin thioesterase otubain-like, putative, expressed |
| *GRMZM2G121928* | 4 | 60,384,134 | 60,384,965 |  |  |  |  |  |  |
| *GRMZM2G121942* | 4 | 60,386,939 | 60,393,456 | *LOC_Os08g34480* | *AT4G14342.1* |  |  |  | splicing factor 3B subunit 5, putative, expressed |
| *GRMZM2G370863* | 4 | 85,758,755 | 85,759,432 | *LOC_Os09g29130* | *AT3G28917.1* | *ZHD19* |  | ZF-HD-transcription factor 19 | ZF-HD protein dimerisation region containing protein, expressed |
| *GRMZM2G459230* | 4 | 118,286,308 | 118,287,793 | *LOC_Os01g01340* | *AT3G26740.1* |  |  |  | light-induced protein 1-like, putative, expressed |
| *GRMZM2G150024* | 4 | 124,660,672 | 124,667,062 | *LOC_Os04g38480* | *AT1G34210.1* | *SERK3* |  | somatic embryogenesis receptor-like kinase3 | BRASSINOSTEROID INSENSITIVE 1-associated receptor kinase 1 precursor, putative, expressed |
| *GRMZM2G157016* | 4 | 125,125,002 | 125,131,423 | *LOC_Os11g04954* | *AT3G22880.1* |  |  |  | DNA repair protein Rad51, putative, expressed |
| *GRMZM2G063792* | 4 | 131,279,153 | 131,282,696 | *LOC_Os12g27520* | *AT4G24740.1* |  |  |  | serine/threonine-protein kinase AFC2, putative, expressed |
| *GRMZM2G072690* | 4 | 133,480,125 | 133,485,253 | *LOC_Os02g39070* | *AT1G05785.1* |  |  |  | vesicle transport protein GOT1B, putative, expressed |
| *GRMZM5G864784* | 4 | 136,066,967 | 136,069,031 |  |  |  |  |  |  |
| *GRMZM5G806469* | 4 | 136,069,551 | 136,070,238 |  |  |  |  |  |  |
| *GRMZM2G134752* | 4 | 147,679,277 | 147,680,759 | *LOC_Os02g42820* | *AT3G61230.1* | *LIMTF8* |  | LIM-transcription factor 8 | OsPLIM2a - LIM domain protein, putative actin-binding protein and transcription factor, expressed |
| *GRMZM2G398755* | 4 | 148,257,670 | 148,265,342 | *LOC_Os05g44310* | *AT4G04910.1* |  |  |  | vesicle-fusing ATPase, putative, expressed |
| *GRMZM2G392975* | 4 | 153,654,423 | 153,658,268 | *LOC_Os02g44630* | *AT4G00430.1* | *PIP1D* |  | plasma membrane intrinsic protein1 | aquaporin protein, putative, expressed |
| *GRMZM2G094705* | 5 | 191,171,285 | 191,180,578 | *LOC_Os02g43760* | *AT4G17510.1* | *UCH3* |  |  | ubiquitin carboxyl-terminal hydrolase, family 1, putative, expressed |
| *GRMZM2G094768* | 5 | 191,180,976 | 191,183,011 | *LOC_Os02g43770* | *AT3G60320.1* |  |  |  | DUF630/DUF632 domains containing protein, putative, expressed |
| *GRMZM2G383673* | 5 | 210,689,478 | 210,690,416 |  |  |  |  |  |  |
| *GRMZM2G081371* | 5 | 210,694,091 | 210,694,751 | *LOC_Os02g53070* | *AT5G64930.1* |  |  |  | HYS1, putative, expressed |
| *GRMZM2G081365* | 5 | 210,698,184 | 210,698,352 | *LOC_Os02g53070* |  |  |  |  | HYS1, putative, expressed |
| *GRMZM2G135498* | 6 | 107,993,578 | 107,997,950 | *LOC_Os06g09630* | *AT5G46290.1* |  |  |  | 3-oxoacyl-synthase, putative, expressed |
| *GRMZM2G059151* | 6 | 115,372,536 | 115,378,936 | *LOC_Os06g13810* | *AT1G12000.1* | *PFK1* |  | phosphofructose kinase1 | pyrophosphate--fructose 6-phosphate 1-phosphotransferase subunit beta, putative, expressed |
| *GRMZM2G117057* | 6 | 121,720,043 | 121,721,582 | *LOC_Os06g30370* | *AT1G18100.1* | *PEBP11* |  | phosphatidylethanolamine-binding protein11 | osMFT1 MFT-Like1 homologous to Mother of FT and TFL1 gene; contains Pfam profile PF01161: Phosphatidylethanolamine-binding protein, expressed |
| *GRMZM2G002473* | 6 | 128,703,781 | 128,708,037 | *LOC_Os08g39220* | *AT1G21270.1* |  |  |  | OsWAK75 - OsWAK receptor-like protein kinase, expressed |
| *GRMZM5G831025* | 6 | 130,147,524 | 130,148,121 |  |  |  |  |  |  |
| *GRMZM5G828487* | 6 | 130,148,760 | 130,149,582 | *LOC_Os01g09470* | *AT3G16490.1* |  |  |  | IQ calmodulin-binding motif family protein, expressed |
| *GRMZM2G088501* | 6 | 134,052,641 | 134,061,915 | *LOC_Os05g18470* | *AT4G08690.1* |  |  |  | CRAL/TRIO domain containing protein, expressed |
| *GRMZM2G036436* | 6 | 134,071,895 | 134,073,982 | *LOC_Os05g16300* | *AT5G05840.1* |  |  |  | expressed protein |
| *GRMZM2G325804* | 6 | 138,372,260 | 138,380,446 | *LOC_Os05g30010* | *AT3G06880.2* |  |  |  | WD domain, G-beta repeat domain containing protein, expressed |
| *GRMZM2G302912* | 6 | 138,844,999 | 138,846,317 | *LOC_Os05g29676* | *AT1G72200.1* |  |  |  | RING-H2 finger protein, putative, expressed |
| *GRMZM2G106265* | 6 | 140,043,293 | 140,046,488 | *LOC_Os08g23440* | *AT1G30450.1* |  |  |  | amino acid permease family protein, putative, expressed |
| *GRMZM2G113276* | 6 | 140,056,133 | 140,067,126 | *LOC_Os08g23440* | *AT1G30450.1* |  |  |  | amino acid permease family protein, putative, expressed |
| *AC213884.3_FG001* | 6 | 140,654,815 | 140,658,705 | *LOC_Os05g28290* | *AT2G30060.1* |  |  |  | ranBP1 domain containing protein, expressed |
| *GRMZM6G157558* | 6 | 141,037,374 | 141,038,317 | *LOC_Os05g27870* | *AT5G58450.1* |  |  |  | expressed protein |
| *GRMZM2G020929* | 6 | 141,037,421 | 141,038,338 | *LOC_Os05g27870* | *AT5G58450.1* |  |  |  | expressed protein |
| *GRMZM2G115255* | 6 | 141,047,201 | 141,050,134 | *LOC_Os05g27870* | *AT5G58450.1* |  |  |  | expressed protein |
| *GRMZM2G084719* | 6 | 141,493,173 | 141,497,370 | *LOC_Os05g28500* | *AT2G30100.1* |  |  |  | ubiquitin family protein, putative, expressed |
| *GRMZM2G381318* | 6 | 146,413,281 | 146,414,549 |  |  |  |  |  |  |
| *GRMZM2G080898* | 6 | 146,415,531 | 146,417,114 | *LOC_Os05g33760* | *AT2G30780.1* |  |  |  | PPR repeat domain containing protein, putative, expressed |
| *GRMZM2G080940* | 6 | 146,421,679 | 146,425,974 | *LOC_Os05g33820* | *AT1G10740.1* |  |  |  | lipase, putative, expressed |
| *GRMZM2G353209* | 6 | 149,134,586 | 149,138,083 | *LOC_Os05g37690* | *AT2G39940.1* |  |  |  | OsFBL23 - F-box domain and LRR containing protein, expressed |
| *GRMZM2G171139* | 6 | 149,912,055 | 149,914,664 | *LOC_Os05g37250* | *AT2G27690.1* |  |  |  | cytochrome P450, putative, expressed |
| *GRMZM2G420899* | 8 | 2,310,806 | 2,311,144 | *LOC_Os11g38040* | *AT5G06430.1* |  |  |  | expressed protein |
| *GRMZM2G004157* | 9 | 149,545,036 | 149,547,415 | *LOC_Os05g19970* | *AT3G62240.1* |  |  |  | ZOS5-06 - C2H2 zinc finger protein, expressed |
| *GRMZM2G004119* | 9 | 149,547,639 | 149,549,020 | *LOC_Os05g19970* | *AT3G62240.1* |  |  |  | ZOS5-06 - C2H2 zinc finger protein, expressed |
| *GRMZM2G456241* | 9 | 149,570,706 | 149,575,220 | *LOC_Os05g19970* | *AT3G62240.1* |  |  |  | ZOS5-06 - C2H2 zinc finger protein, expressed |
| *GRMZM2G152815* | 9 | 149,629,715 | 149,634,020 | *LOC_Os05g19970* | *AT3G62240.1* |  |  |  | ZOS5-06 - C2H2 zinc finger protein, expressed |

Supplementary Table 4. Quantitative trait loci (QTLs) for 10 yield-related traits of a maize DH population growing in three different environments

| **QTL** | **Chr** | **Position (cM)** | **Estimated interval (bp)** | **15TZ-spring** | |  | **15TZ-summer** | |  | **15XTS** |  |  |
| --- | --- | --- | --- | --- | --- | --- | --- | --- | --- | --- | --- | --- |
|  |  |  |  | **LOD** | **%PVE** | **Add** | **LOD** | **%PVE** | **Add** | **LOD** | **%PVE** | **Add** |
| Rows per ear |  |  |  |  |  |  |  |  |  |  |  |  |
| *qrpe1* | 1 | 18.5 | 5930268-7924066 | 2.9254 | 5.3904 | -0.3235 |  |  |  |  |  |  |
| *qrpe2-1* | 2 | 61.5 | 18752480-19165918 |  |  |  |  |  |  | 5.2721 | 7.4741 | 0.4367 |
| *qrpe2-2* | 2 | 64 | 19165918-22651971 |  |  |  | 5.5422 | 9.9422 | 0.5154 |  |  |  |
| *qrpe2-3* | 2 | 68.5 | 28664054-28952271 | 2.6438 | 4.8177 | 0.3066 |  |  |  |  |  |  |
| *qrpe4-1* | 4 | 105 | 18692542-19941128 |  |  |  |  |  |  | 8.2564 | 12.2241 | -0.5585 |
| *qrpe4-2* | 4 | 103 | 20660072-22868500 |  |  |  | 4.7676 | 8.6425 | -0.4803 |  |  |  |
| *qrpe4-3* | 4 | 96.5 | 31497505-33000282 | 5.7507 | 10.8234 | -0.4578 |  |  |  |  |  |  |
| *qrpe4-4* | 4 | 51.5 | 180346673-181177073 |  |  |  | 2.6086 | 4.5045 | -0.3579 |  |  |  |
| *qrpe5* | 5 | 227.5 | 213659859-213995510 | 2.9413 | 5.358 | -0.3236 |  |  |  |  |  |  |
| *qrpe8-1* | 8 | 42 | 16787762-17234908 |  |  |  |  |  |  | 3.2265 | 4.4767 | 0.344 |
| *qrpe8-2* | 8 | 135.5 | 171279999-173059278 | 2.712 | 4.9851 | 0.314 |  |  |  |  |  |  |
| *qrpe9* | 9 | 43 | 15890657-16088923 |  |  |  |  |  |  | 4.5305 | 6.3704 | 0.4098 |
| *qrpe10* | 10 | 49 | 42129316-95744014 |  |  |  |  |  |  | 3.4781 | 4.893 | -0.3606 |
| Kernel weight per ear | |  |  |  |  |  |  |  |  |  |  |  |
| *qkwpe1-1* | 1 | 97.5 | 174530056-179076839 | 8.4483 | 17.3949 | -10.2919 |  |  |  |  |  |  |
| *qkwpe1-2* | 1 | 101 | 182970995-183860432 |  |  |  | 13.4864 | 21.3167 | -13.7843 |  |  |  |
| *qkwpe4* | 4 | 102.5 | 20660072-22868500 |  |  |  |  |  |  | 4.9417 | 8.5058 | -8.9371 |
| Kernel number per row | |  |  |  |  |  |  |  |  |  |  |  |
| *qknpr1-1* | 1 | 49 | 24296966-25508129 |  |  |  | 3.3136 | 3.3879 | 1.1092 |  |  |  |
| *qknpr1-2* | 1 | 99.25 | 175527798-182970995 |  |  |  | 11.9361 | 13.304 | -2.37 | 5.2793 | 8.1432 | -1.927 |
| *qknpr1-3* | 1 | 102 | 182970995-183860432 | 4.9862 | 8.5713 | -1.5956 |  |  |  |  |  |  |
| *qknpr1-4* | 1 | 181.25 | 263692231-285836685 | 2.6572 | 5.1 | 1.1587 | 3.1498 | 3.1722 | 1.1022 |  |  |  |
| *qknpr5-1* | 5 | 140.5 | 13323624-29257275 |  |  |  |  |  |  | 4.3997 | 6.6795 | 1.6237 |
| *qknpr5-2* | 5 | 161 | 109300417-130142038 |  |  |  | 4.4285 | 4.5223 | 1.2682 |  |  |  |
| Volume weight | |  |  |  |  |  |  |  |  |  |  |  |
| *qvw1* | 1 | 101.75 | 182970995-183860432 | 5.9821 | 15.4974 | -40.4216 | 7.0172 | 18.2106 | -55.4796 |  |  |  |
| *qvw4* | 4 | 104 | 20625756-20660072 |  |  |  |  |  |  | 4.9249 | 11.3603 | -44.095 |
| *qvw5* | 5 | 11.5 | 3534277-5269648 |  |  |  |  |  |  | 2.5437 | 6.4167 | 34.3899 |
| Water content | |  |  |  |  |  |  |  |  |  |  |  |
| *qwc5* | 5 | 155 | 30095502-31796005 |  |  |  | 3.5338 | 13.1143 | -0.1569 |  |  |  |
| Ear length |  |  |  |  |  |  |  |  |  |  |  |  |
| *qel1-1* | 1 | 97.5 | 174530056-179076839 |  |  |  | 10.1218 | 14.9072 | -0.7771 | 4.7708 | 6.5556 | -0.5745 |
| *qel1-2* | 1 | 104.5 | 190473285-193221577 | 5.7041 | 10.2205 | -0.6577 |  |  |  |  |  |  |
| *qel1-3* | 1 | 148.5 | 252936690-255832047 |  |  |  | 2.5187 | 3.4328 | 0.3614 |  |  |  |
| *qel1-4* | 1 | 188.5 | 263692231-285836685 | 3.6767 | 7.0455 | 0.5256 |  |  |  | 4.5558 | 6.3193 | 0.5359 |
| *qel2-1* | 2 | 124 | 204881569-205813273 |  |  |  |  |  |  | 3.5558 | 5.0361 | 0.4658 |
| *qel2-2* | 2 | 127 | 205813273-207010117 |  |  |  | 2.5116 | 3.6183 | 0.3519 |  |  |  |
| *qel3-1* | 3 | 107 | 2125451-2775846 |  |  |  | 2.9787 | 4.228 | -0.4058 |  |  |  |
| *qel3-2* | 3 | 100 | 2838953-3652892 |  |  |  |  |  |  | 5.4493 | 7.5768 | -0.5896 |
| *qel6* | 6 | 94 | 161766171-162264683 |  |  |  | 2.7553 | 3.7872 | 0.3649 |  |  |  |
| *qel8-1* | 8 | 37 | 12476088-14650891 |  |  |  | 5.1829 | 7.6877 | -0.5229 |  |  |  |
| *qel8-2* | 8 | 58.5 | 102759728-115738864 | 4.3235 | 7.5629 | -0.5351 |  |  |  |  |  |  |
| Bare top length | |  |  |  |  |  |  |  |  |  |  |  |
| *qbtl1* | 1 | 103.5 | 183860432-189750803 |  |  |  | 4.8101 | 10.1189 | 0.2666 |  |  |  |
| *qbtl7* | 7 | 76.5 | 123636460-123760484 |  |  |  |  |  |  | 4.9768 | 7.9042 | -0.2417 |
| *qbtl8* | 8 | 52 | 22850793-22879172 |  |  |  |  |  |  | 3.179 | 4.9335 | 0.199 |
| *qbtl9* | 9 | 137 | 139380687-141014967 |  |  |  |  |  |  | 5.9029 | 9.4482 | 0.2621 |
| Ear weight |  |  |  |  |  |  |  |  |  |  |  |  |
| *qew1-1* | 1 | 80.5 | 70915224-71021697 |  |  |  |  |  |  | 2.6162 | 5.3769 | -1.8217 |
| *qew1-2* | 1 | 97 | 174263305-174530056 | 6.7289 | 13.6909 | -1.9567 | 14.3114 | 19.2741 | -2.1715 |  |  |  |
| *qew2* | 2 | 3 | 885049-2112963 |  |  |  | 3.0314 | 3.7456 | -0.887 |  |  |  |
| *qew5* | 5 | 173.5 | 190282997-191306526 |  |  |  |  |  |  | 3.1075 | 6.4216 | -1.9951 |
| *qew9* | 9 | 165.5 | 147643829-148578237 | 2.5226 | 4.8835 | -1.0681 |  |  |  |  |  |  |
| Kernel number per ear | |  |  |  |  |  |  |  |  |  |  |  |
| *qknpe1-1* | 1 | 97.5 | 174530056-179076839 |  |  |  | 4.1812 | 9.1678 | -27.267 | 3.4205 | 6.4753 | -28.287 |
| *qknpe1-2* | 1 | 100.5 | 180690508-182970995 |  |  |  | 4.8994 | 7.4774 | -28.8031 |  |  |  |
| *qknpe1-3* | 1 | 186.5 | 263692231-285836685 |  |  |  | 2.9899 | 4.4708 | 21.2836 |  |  |  |
| *qknpe4-1* | 4 | 102.5 | 20660072-22868500 |  |  |  |  |  |  | 3.5737 | 6.9587 | -27.073 |
| *qknpe4-2* | 4 | 93.5 | 36777690-37218419 |  |  |  | 2.8831 | 4.3633 | -20.1374 |  |  |  |
| *qknpe10* | 10 | 24 | 4204494-4408153 |  |  |  | 3.0623 | 4.5861 | -20.8443 |  |  |  |
| Hundred grain weight | |  |  |  |  |  |  |  |  |  |  |  |
| *qhgw1-1* | 1 | 104.5 | 190473285-193221577 |  |  |  | 12.618 | 22.0609 | -2.1784 |  |  |  |
| *qhgw1-2* | 1 | 106 | 193221577-194392697 | 4.7248 | 10.1753 | -1.5016 |  |  |  |  |  |  |
| *qhgw10* | 10 | 33 | 5436299-8925180 |  |  |  | 3.2442 | 5.1319 | 0.9761 |  |  |  |

Supplementary Table 5. The cumulative additive effect of QTL for rows per ear in all 15 X lines and 2 parents.

| Sample Name | Cumulative Additive Effect | QTL Name | | | | | | | | | | | | |
| --- | --- | --- | --- | --- | --- | --- | --- | --- | --- | --- | --- | --- | --- | --- |
|  |  |  |  |  |  |  |  |  |  |  |  |  |  |  |
|  |  |  |  |  |  |  |  |  |  |  |  |  |  |  |
|  |  | *qrpe1* | *qrpe2-1* | *qrpe2-2* | *qrpe2-3* | *qrpe4-1* | *qrpe4-2* | *qrpe4-3* | *qrpe4-4* | *qrpe5* | *qrpe8-1* | *qrpe8-2* | *qrpe9* | *qrpe10* |
| PH4CV | -0.5357 | - | + | + | + | - | - | - | - | - | + | + | + | - |
| PH6WC | 0.5357 | + | - | - | - | + | + | + | + | + | - | - | - | + |
| Jing72464 | 3.5941 | + | - | + | + | + | + | + | + | + | + | + | + | - |
| Jing724 | 2.8783 | + | - | + | + | + | + | + | - | + | + | + | + | - |
| Jing464 | 2.8783 | + | - | + | + | + | + | + | - | + | + | + | + | - |
| Jing725 | 2.8783 | + | - | + | + | + | + | + | - | + | + | + | + | - |
| JingMC01 | 2.4767 | - | + | + | + | + | + | + | - | + | + | - | + | - |
| D9B | 2.3727 | + | + | + | + | + | + | + | + | - | + | - | - | - |
| D9H | 2.3727 | + | + | + | + | + | + | + | + | - | + | - | - | - |
| JingDH3345 | 1.7619 | + | + | + | + | - | - | + | + | + | + | - | + | - |
| DH382 | 1.4283 | - | + | + | + | - | + | + | + | - | + | - | + | - |
| YuA9241 | 1.3275 | + | - | - | - | + | + | + | - | + | + | - | + | + |
| 91277 | 0.5767 | - | - | - | - | + | + | + | + | + | + | - | - | + |
| B547 | 0.3991 | - | + | + | + | - | - | + | - | + | + | - | + | - |
| Jing4055 | 0.1583 | + | + | + | + | - | - | - | + | + | - | - | + | - |
| Jing88 | 0.1583 | + | + | + | + | - | - | - | + | + | - | - | + | - |
| X24621 | -2.1097 | - | - | - | - | - | - | + | + | - | - | + | + | - |

Supplementary Table 6. LOD, PVE and additive effect of the QTLs for rows per ear.

| **QTL Name** | **LOD** | **PVE(%)** | **Add** |
| --- | --- | --- | --- |
| *qrpe1* | 2.9254 | 5.3904 | -0.324 |
| *qrpe2-1* | 5.2721 | 7.4741 | 0.4367 |
| *qrpe2-2* | 5.5422 | 9.9422 | 0.5154 |
| *qrpe2-3* | 2.6438 | 4.8177 | 0.3066 |
| *qrpe4-1* | 8.2564 | 12.2241 | -0.559 |
| *qrpe4-2* | 4.7676 | 8.6425 | -0.48 |
| *qrpe4-3* | 5.7507 | 10.8234 | -0.458 |
| *qrpe4-4* | 2.6086 | 4.5045 | -0.358 |
| *qrpe5* | 2.9413 | 5.358 | -0.324 |
| *qrpe8-1* | 3.2265 | 4.4767 | 0.344 |
| *qrpe8-2* | 2.712 | 4.9851 | 0.314 |
| *qrpe9* | 4.5305 | 6.3704 | 0.4098 |
| *qrpe10* | 3.4781 | 4.893 | -0.361 |

# Supplementary Figures


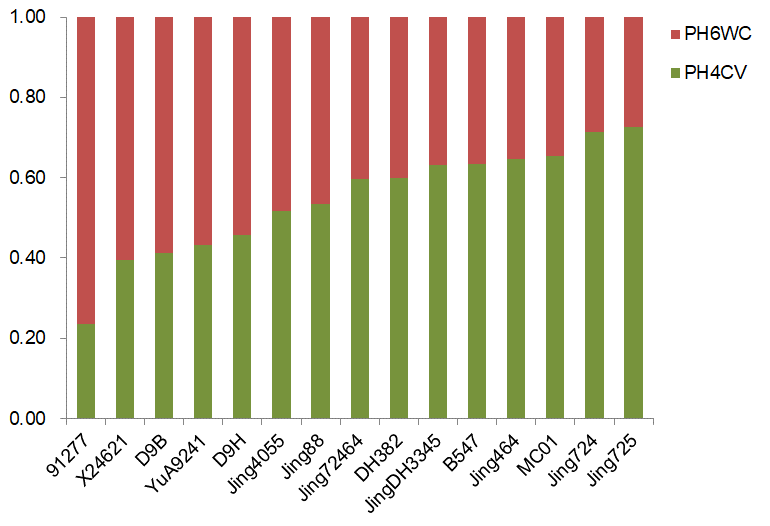


Supplementary Figure 1. The percentage of genomic IBD regions in 15 elite X lines

##
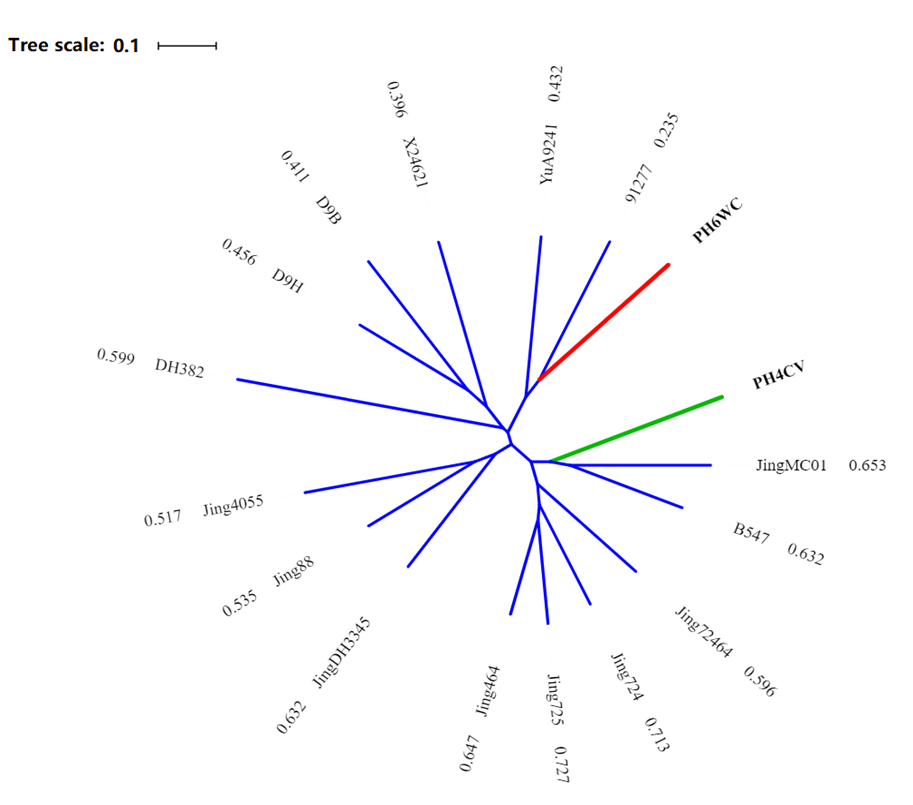


Supplementary Figure 2. Neighbor-joining phylogenetic tree of 15 X lines and their parents.


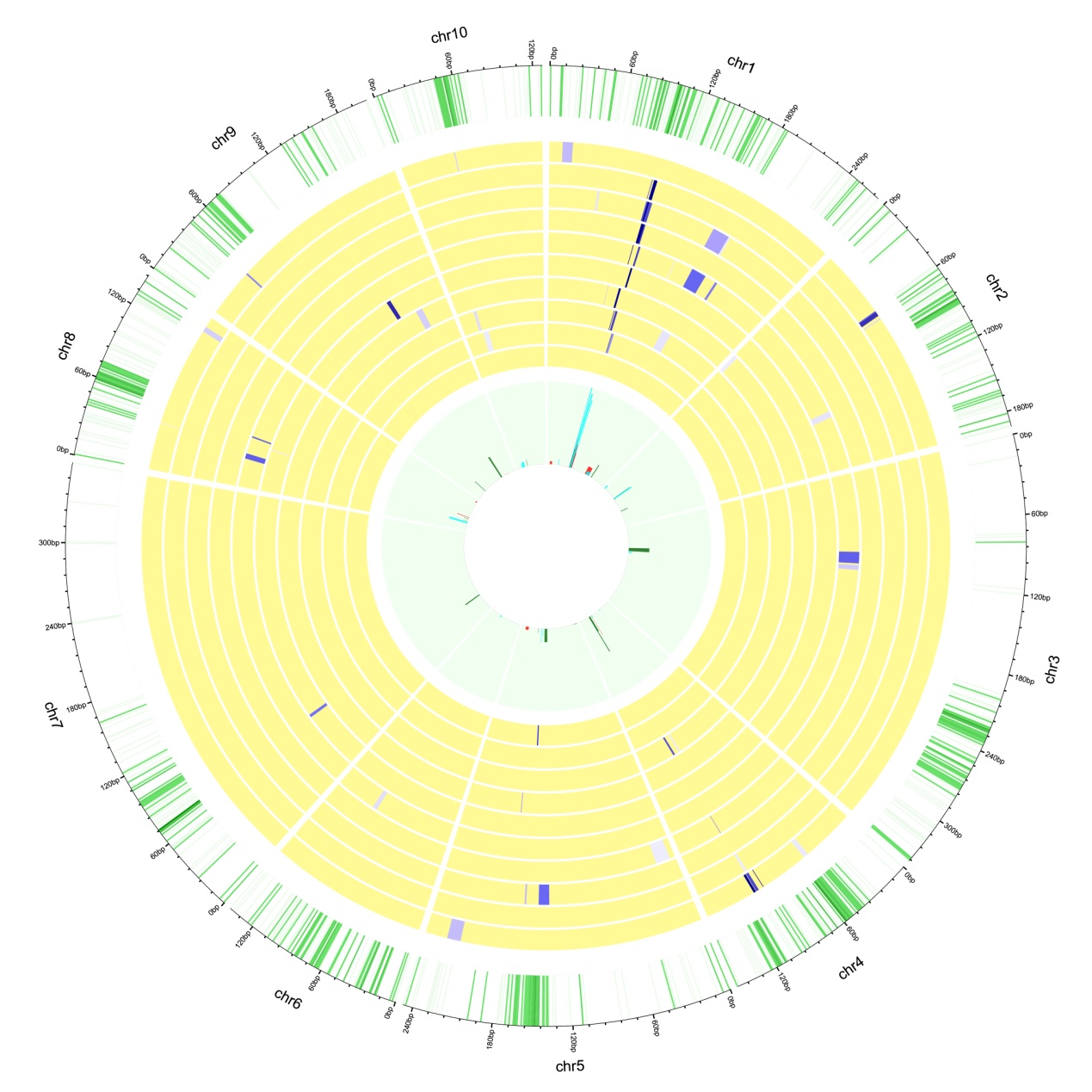


Supplementary Figure 3. Overview of quantitative trait loci (QTLs) associated with 10 important agronomic traits. The outer histogram represents the genetic linkage map for the DH population derived from PH4CV × PH6WC. The inner heatmap indicates the QTLs in the genome, with the color intensity reflecting the PVE value.

Supplementary Figure 4. Functional annotation of genes from the ICRs and SSWs in PH4CV and PH6WC.
